# Toward Predicting Impact of Common Genetic Variants on Schizophrenia Clinical Responses With Antipsychotics: A Quantitative System Pharmacology Study

**DOI:** 10.3389/fnins.2021.738903

**Published:** 2021-09-29

**Authors:** Athan Spiros, Hugo Geerts

**Affiliations:** ^1^In Silico Biosciences, Berwyn, PA, United States; ^2^Certara QSP, Canterbury, United Kingdom

**Keywords:** pharmacodynamics, system pharmacology, dopamine – physiology, serotonin physiology, cognition, motor symptom

## Abstract

CNS disorders are lagging behind other indications in implementing genotype-dependent treatment algorithms for personalized medicine. This report uses a biophysically realistic computer model of an associative and dorsal motor cortico-striatal-thalamo-cortical loop and a working memory cortical model to investigate the pharmacodynamic effects of COMTVal158Met rs4680, 5-HTTLPR rs 25531 s/L and D2DRTaq1A1 genotypes on the clinical response of 7 antipsychotics. The effect of the genotypes on dopamine and serotonin dynamics and the level of target exposure for the drugs was calibrated from PET displacement studies. The simulations suggest strong gene-gene pharmacodynamic interactions unique to each antipsychotic. For PANSS Total, the D2DRTaq1 allele has the biggest impact, followed by the 5-HTTLPR rs25531. The A2A2 genotype improved efficacy for all drugs, with a more complex outcome for the 5-HTTLPR rs25531 genotype. Maximal range in PANSS Total for all 27 individual combinations is 3 (aripiprazole) to 5 points (clozapine). The 5-HTTLPR L/L with aripiprazole and risperidone and the D2DRTaq1A2A2 allele with haloperidol, clozapine and quetiapine reduce the motor side-effects with opposite effects for the s/s genotype. The COMT genotype has a limited effect on antipsychotic effect and EPS. For cognition, the COMT MM 5-HTTLPR L/L genotype combination has the best performance for all antipsychotics, except clozapine. Maximal difference is 25% of the total dynamic range in a 2-back working memory task. Aripiprazole is the medication that is best suited for the largest number of genotype combinations (10) followed by Clozapine and risperidone (6), haloperidol and olanzapine (3) and quetiapine and paliperidone for one genotype. In principle, the platform could identify the best antipsychotic treatment balancing efficacy and side-effects for a specific individual genotype. Once the predictions of this platform are validated in a clinical setting the platform has potential to support rational personalized treatment guidance in clinical practice.

## Introduction

Personalized medicine is rapidly becoming more accepted in various disease indications, especially oncology. However, the concept has not been embraced to the same extent in CNS disorders where the “blockbuster” idea of “one size fits all” has been the strategy of drug discovery and development for many decades. Recent attempts to better tailor treatment paradigms to individual schizophrenia patients have relied mostly on analysis of Big Data sets with identifying genetic variants associated both with pharmacodynamics and metabolism of psycho-active drugs (Rigby et al., 2019). In fact, bringing in such considerations early in the treatment plan has been shown to improve overall outcome (Rigby et al., 2019). For example, five SNP have been associated with a clinical response for iloperidone (Lavedan et al., 2008). Other efforts to identify genotypes associated with clinical response have been performed in bipolar disorder and depression (Maciukiewicz et al., 2018).

The effect of common genotype variants, for example the COMTVal158Met (Lachman et al., 1996), 5-HTTLPR rs 25531 s/L (Heils et al., 1995) and D2DRTaq1A1 (Neville et al., 2004) has not been studied in great detail (see Tables 1, 2 for the reported studies). A few studies have looked at epistasis, i.e., how genotypes interact with each other in a pharmacodynamic way. As many antipsychotic drugs have a complex pharmacology it is expected that this would add even more complexity.

**TABLE 1:** Clinical studies on the effect of COMTVal158Met, 5-HTTLPR, and DRD2Taq1A genotypes on antipsychotic clinical response to PANSS and motor side-effects in schizophrenia.

Subjects	Genotype(s)	Drug(s)	Outcome	References
93 subjects on BPRS	COMT DRD4	clozapine	No effect of either gene alone; interaction between DRD4 and COMT	Rajagopal et al., 2018
107 subjects on PANSS Negative	COMT, 5HT1A (−1019 C/G)	clozapine	Greater improvement with COMT VV genotype	Bosia et al., 2015
240 male subjects SAS	COMT, DRD2Taq1A	haloperidol	COMT MV had 1.7 times greater risk for EPS than other COMT genotypes	Zivkovic et al., 2013
329 Caucasian subjects and CGI-improvement	COMTVal158Met and D2DRTaq1A	risperidone, olanzapine	No effect of COMT and DRD2	Vehof et al., 2012
116 Swedish patients and EPS symptoms	DRD2Taq1A	Different drugs	A1 allele has greater EPS if they were treated on drugs with strong D2 antagonism	Alenius et al., 2008
119 patients and EPS symptoms	DRD2Taq1A and 5-HTT LPR	Different drugs	A1 allele has greater EPS; no effect of 5-HTTLPR	Guzey et al., 2007
56 patients and BPRS	5-HTTLPR rs25531	Haloperidol and Risperidone	S allele associated with lower improvement on BPRS	Dolzan et al., 2008
141 Korean patients on PANSS and EPS	5-HTTLPR rs25531	Different atypical drugs	No effect of genotypes on PANSS or EPS side effects	Lee et al., 2009
90 Korean treatment resistant patients	5-HTTLPR rs25531	clozapine	No effect on BPRS outcome	Tsai et al., 2000

**TABLE 2:** Clinical studies on the effect of COMTVal158Met genotype on cognitive outcome in schizophrenia.

Subjects	Outcome	References
145 schizophrenia with Computerized Cognitive Remediation	COMT Met allele significant improvement	Lindenmayer et al., 2015
135 Chronic schizophrenia with cannabis use	COMT Met better on cognition	Bosia et al., 2019
100 schizophrenia patients	COMT Met better on social cognition	Tylec et al., 2017
59 Brazilian schizophrenia outpatients	COMT Met worse on executive function; in DRD3 Ser/Ser genotype	Loch et al., 2015
118 Japanese schizophrenia patients	COMT Met superior in various executive functions	Tsuchimine et al., 2013
27 schizophrenia patients	COMT Met worse for paced serial order recall	Hill et al., 2013
114 Spanish schizophrenia patients and 74 relatives	COMT Met better in Dot Pattern Expectancy Test, no effect of genotype on Matrics-scale	Lopez-Garcia et al., 2013
87 schizophrenia patients on Wisconsin Card Sorting Test	COMT Val/Met performed worse than homozygotes at baseline; no effect of genotype on improvement with Cognitive Remediation therapy	Greenwood et al., 2011
67 schizophrenia patients Stroop test	COMT Met better in tasks with cognitive stability; no effect of genotype on tasks with cognitive flexibility	Rosa et al., 2010
364 schizophrenia patients	No effect of COMT genotype on RBANS	Dickerson et al., 2007
130 Spanish patients in first episode non-affective psychosis	No effect on cognitive performance	Mata et al., 2008

*Unfortunately, no data are available on the nature of antipsychotics in these studies.*

Because of this tremendous complex interaction, it can be a challenge to derive these insights from existing patient datasets as each patient with their specific drug-dose combination and genotype combination is basically a unique subject. Traditional statistical methods often lack the granularity to account for these interactions, as they often need to aggregate data in various classes. In addition, clinical response is often modulated by non-genetic factors, such as other comedications and smoking status. Machine-learning approaches need a robust training set, but it is hard to envision generalizing outcomes to different antipsychotics with quite complex pharmacology or with dopaminergic and serotonergic genotypes and combinations thereof. However, it is possible to derive associations between SNPs and clinical response in schizophrenia; examples include the MEGF10, TNIK, SLC1A1, PCDH7, CNTNAP5, each of them affecting responses to different antipsychotics in a different way in a Chinese population (Yu et al., 2018).

A possible alternative is to use advanced computer modeling of humanized brain circuits based on formalized domain expertise to predict the interaction of the pharmacology of different antipsychotics with these common genotype variants for which clinical data (imaging) are available and then test the predictions on an individual patient level in large databases. Quantitative Systems Pharmacology (QSP) is a biophysically realistic computer model of the neuronal activity in complex neuronal networks informed by human neuro-anatomy and neurophysiology. The platform calculates the firing dynamics of the closed cortico-striatal-thalamo-cortical loop, more specifically the information content in the thalamic reticular nucleus (Pratt and Morris, 2015) is constrained by calibrating with historical clinical trials both for efficacy (PANSS Total) and motor side-effects (EPS) and has shown predictive validity in schizophrenia (Geerts et al., 2012; Liu et al., 2014). This approach has also been used to prospectively predict an unexpected clinical outcome for an as yet untested pro-cognitive target (Nicholas et al., 2013) and to explore hypotheses about the failure of amyloid-modulating agents in Alzheimer’s disease (Geerts et al., 2018; Geerts and Spiros, 2020).

In this study we simulate the effect of the COMTVal158Met, the 5-HTTLPR rs25531 L/s promotor region and the DRD2Taq1A1 allele in all possible combinations for seven commonly used antipsychotics, aripiprazole, risperidone, clozapine, haloperidol, olanzapine, paliperidone and quetiapine on efficacy (change in PANSS Total), motor side-effects (EPS liability) and cognitive outcome. We focused on these three genotypes because there were human imaging data available on the effects of these genotypes variants on the relevant neurotransmitter circuits. It is to be noted that this platform is a hypothesis generating engine and that the results of this simulation, once validated, could help identify the ‘best’ antipsychotic in terms of benefit over risk for any individual configuration of these three genotypes.

## Materials and Methods

### Quantitative Systems Pharmacology Model for PANSS Total

The QSP model of the closed cortico-striatal-thalamo-cortical circuit has been described in detail elsewhere (Terman et al., 2002) and adapted for the pathology of schizophrenia (see Supplementary Information 3 for a detailed description of the cortico-striatal-thalamocortical circuit). The platform basically models a neuronal circuit of the basal ganglia. D_1_R + and D_2_ R + striatal medium spiny neurons are driven by cortical input, modulated by dopamine afferents from the ventral tegmentum area and projecting in the direct and indirect pathway, respectively. Over 30 CNS targets are implemented based on their known localization and their intracellular coupling to voltage- or ligand gated ion channels. Schizophrenia pathology is introduced based a quantitative analysis of human imaging studies on hyperdopaminergic state in the striatum (Abi-Dargham et al., 2000) and hypodopaminergic pathology in the cortex (Meyer-Lindenberg et al., 2002) together with a cortical glutamatergic and GABA-ergic deficit and an increase in noise level (Geerts et al., 2013).

The Shannon-type information entropy (Strong et al., 1998) in the Thalamic Reticular Nucleus as a measure of signal bandwidth correlates very well with clinical outcomes of antipsychotics on PANSS Total and is used as a proxy for change in PANSS Total (Spiros et al., 2017; see Supplementary Information 6). Interestingly, recent intracranial studies in human and primates suggest that humans prefer efficiency over robustness and that their neuronal activity is better represented by a Shannon-type of information content (Pryluk et al., 2019).

### Quantitative Systems Pharmacology Model for Extrapyramidal Symptoms

The QSP model of motor symptoms has been described before and calibrated (Roberts et al., 2016) extensively both with the reported Extra-Pyramidal Symptoms (EPS) side-effect with antipsychotic medication as well as a number of therapeutic interventions in Parkinson’s disease (see also Supplementary Information Section 4 for a detailed description of the motor circuit). The system uses the same cortico-striatal-thalamo-cortical circuit as the PANSS Total circuit with a focus on the motor loop and the readout of the power spectrum of local field potentials in the Subthalamic Nucleus. From clinical studies with deep-brain stimulation in Parkinson’s patients (Little et al., 2013), the ratio of beta-over gamma power of these local field potentials is proportional to a measure of clinical rigidity and bradykinesia.

### Quantitative Systems Pharmacology Model for Cognitive Impairment in Schizophrenia

The QSP model for cognition has been extensively described before (Roberts et al., 2012; Nicholas et al., 2013). Basically, the model consists of a biophysically realistic network of 80 Prefrontal Cortex pyramidal glutamatergic and 30 GABAergic interneurons, with the effects of dopaminergic, serotonergic, noradrenergic and cholinergic modulation (see also Supplementary Information 5.1 and 5.2 for a detailed description of the cognitive network). A short stimulus (50 ms) is injected at time = 2 s and the time over which this information can be kept actively in the network without further stimulation is calculated as the working memory span. This parameter can be affected by pathological changes, the impact of genotypes and/or pharmacological interventions. The cortical schizophrenia pathology as mentioned in Section 1, has been calibrated to reflect a decrease in cognitive readout which is 1.5 standard deviations below the average of the normal subjects (Elvevag and Goldberg, 2000). Calibration of the model for cognitive impairment in schizophrenia (see also Supplementary Information 5.3 for a detailed description of the calibration of the cognitive network) was performed on the accuracy of the 2-back working memory in 17 different clinical studies (Geerts et al., 2013).

### Effect of Antipsychotics on Different Receptor Subtypes

The generic receptor model (see also Supplementary Information 1 for a detailed description of the receptor competition model) simulates the competition between neurotransmitters and the active moiety of therapeutic intervention(s) at the level of the postsynaptic receptor (Spiros et al., 2010), based on pre- and postsynaptic physiology of different neurotransmitter systems, but ultimately constrained by human imaging data (Spiros et al., 2010). Schizophrenia pathology affects dopamine dynamics associated and is implemented based on human imaging studies in patients (Abi-Dargham et al., 2000). Intrasynaptic functional concentration of the active moiety of antipsychotics at a specific dose is calculated by simulating quantitative clinical PET imaging displacement studies with specific D_2_-specific radiotracers. This allows us to probe a dose-range for each antipsychotic where changes in target activation can be calculated from the competition with the respective endogenous neurotransmitter.

Absolute PANSS Total was calculated for the standard dose used in clinical practice, according to the label and corresponding to 20 mg aripiprazole (ARI), 200 mg clozapine (CLO), 15 mg olanzapine (OLA), 4 mg risperidone (RIS), 10 mg haloperidol (HAL), 400 mg quetiapine (QUE), and 9 mg paliperidone (PAL). The slope of the dose-response for each antipsychotic was calculated by fitting a linear response for a range between 20 and 140% of this standard dose.

### Implementation of Common Genotype Variants

The same receptor competition model is used to derive the impact of common human genotype variants on the dynamics of specific neurotransmitter systems (see also Supplementary Information 2 for a detailed description of simulating the PET imaging studies). For instance, the COMTVal158Met genotype affects the basal level of dopamine as measured by the displacement of the D_1_R PET radiotracer NNC-112 in healthy unmedicated volunteers (Slifstein et al., 2008). To reproduce these experimental findings, the synaptic half-life of dopamine in the COMTVV case was 100 ms, 130 ms in the COMTMV and 160 ms in the COMTMM case. Similarly, the displacement of the 5-HT_4_ PET tracer [11C]SB207145 is dependent upon the 5-HTTLPR s/l isoform (Fisher et al., 2012), resulting in a half-life of 55 ms for the LL case, 75 ms for the L/s case and 100 ms for the ss case. This is in line with lower basal 5-HT levels for the LL carriers (more binding of the radiotracer as there is less competition), corresponding to higher expression of the 5-HT transporter as found in lymphoblasts (Lesch et al., 1996).

The DRD2Taq1 A1 allele is implemented using a 30% decrease in D_2_R expression (Thompson et al., 1997; Shi et al., 2008), while the homozygote A2 subjects have a 30% increase in D_2_R levels compared to the heterozygous subject.

### Genotype Combinations

In this paper, we study all three possible combinations of the following three genotypes: COMTVal158Met, 5-HTTLPR rs25531 and D2DRTaq1A1 (all together 27 cases). The genotypes are MM, MV, and VV for the COMTVal158Met;, LL, Ls, and ss for the 5-HTTLPR rs25531 and A1A1, A1A2, A2A2, for the D2DRTaq1A1.

A specific genotype combo which consists of one of the three genotypes under consideration is denoted by C/S/D where C is any of the three COMTVal158Met genotypes, S is any of the 5-HTTLPR rs25531 genotypes and D is any of the D2DRTaq1A1 genotypes. For example, the completely heterozygous case is denoted by MV/Ls/A1A2. Distributions are according to the Hardy-Weinberg equilibrium (Lesch et al., 1996).

Many clinical studies are focused on a single genotype. A study on the COMTVal158Met genotype will include COMT MM genotype, denoted by MM/*/* and consisting of all combinations of COMT MM with 5-HTTLPR of LL, Ls, or ss, and D2DRTaq1A1 of A1A1, A1A2, or A2A2 which produces 9 different cases. For each such COMT genotype we then average with the appropriate weighting factor over all the combinations for the two other genotypes, i.e., the heterozygous form having twice as many subjects as each of the homozygous carriers. For example, the triple heterogeneous combination of MV/Ls/A1A2 accounts for 12.5%, while any of the triple homozygotes (for instance VV/LL/A1A1) accounts only for 1.56%.

### PubMed Searches

PubMed search were performed with the following terms “GenotypeX DrugY,” where GenotypeX refers to any of the three studied genotypes and DrugY to any of the 7 antipsychotics (21 searches).

## Results

### Effect of Common Variants on Antipsychotic PANSS Total Dose-Responses

We first simulated the change in PANSS Total for the standard clinical dose vs. baseline of each of the 7 individual antipsychotics (AP) with all 27 combinations of the three genotypes.

Simulations show that the placebo response ranges from −6.1 to −11.1 points, a 5-point difference for the 27 different genotype combinations. For the same antipsychotic at the same dose, the difference between minimal and maximum improvement in anticipated PANSS Total outcome ranges between 3.4 points (aripiprazole) to 5.6 points (clozapine).

With regard to the effects of a single genotype, we simulate group average for individual antipsychotics over one genotype, averaging out the effects of the two other genotypes according to their distribution.

The COMT and 5-HTT LPR rs25531 genotypes have a negligible effect on placebo response (0.8 points difference for COMT with VV/*/* having the greatest response and 1 point for 5-HTTLPR with */LL/* having the greatest response), but the D2DRTaq1A allele has a significant effect on placebo response (a 2.5 point difference with */*/A2A2 having the strongest response).

**FIGURE 1:**
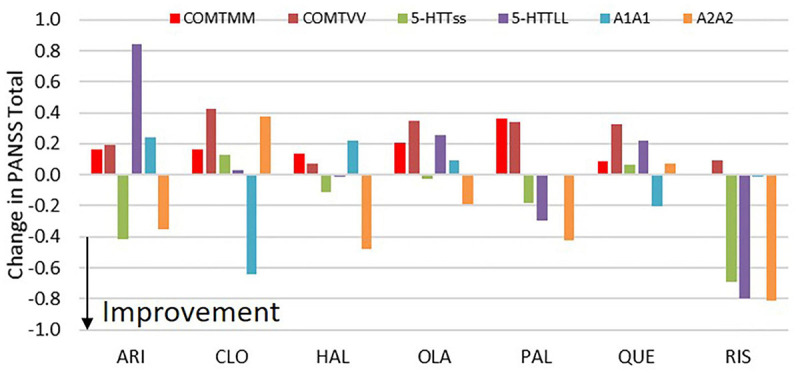
Effect of COMT, 5-HTTLPR rs25531 and D2DRTaq1 genotypes on change in PANSS total vs. placebo for the different antipsychotics at their standard doses. The figure shows the differential effects of the homozygotes (for instance MM/*/* and VV/*/*) vs. the heterozygote subjects (for instance MV/*/*). All results are averaged over the 9 possible combinations of the other two genotypes with the appropriate distribution depending upon the frequency of the genotypes. The biggest effects for the most drugs are seen with the DRD2Taq1 allele, where the */*/A2A2 genotype tends to increase clinical response for drugs with strong D_2_R antagonism, but with an opposite effect for drugs with weak D_2_R antagonism (clozapine and quetiapine). The effect of COMT genotype is limited, but the 5-HTT LPR genotype only has an effect with aripiprazole (*/LL/* worsens outcome) and a complex relationship with risperidone where */Ls/* has the worst outcome.

**FIGURE 2:**
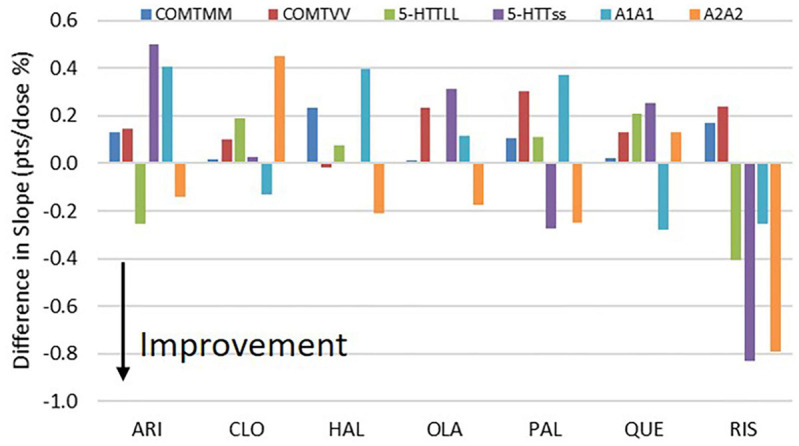
Effect of COMT, 5-HTTLPR rs25531 and D2DRTaq1 genotypes on the slope of the PANSS Total dose-response for the range of doses associated with the different antipsychotics (number of points per 100% change in dose). The figure shows the differential effects of the homozygotes (for instance MM/*/* and VV/*/*) vs. the heterozygote subjects (for instance MV/*/*). All results are averaged over the 9 possible combinations of the other two genotypes with the appropriate distribution depending upon the frequency of the genotypes. Note that positive slope changes indicate that smaller doses are better, while negative slope changes indicate that larger doses are better. Like with the effects on the change in PANSS Total, the biggest effects for the most drugs are seen with the DRD2Taq1 allele, with the */*/A2A2 genotype favorable in drugs with strong D_2_R antagonism, but not in drugs with weak D_2_R antagonism (clozapine and quetiapine). The effect of the COMT genotype is limited, but the 5-HTT LPR genotype has only an effect with aripiprazole and risperidone. The size of the effect is limited; however, for instance the 0.5 effect on slope for */LL/* with aripiprazole translates to a 0.5 point improvement for a dose increase of 100% of the standard dose.

This strong gene-gene interaction (Figures 1, 2) is different for each antipsychotic. For instance, with aripiprazole, the range for PANSS Total response in MM/*/* carriers spans two points, but less than 1 point for */ss/* carriers. This is because the 5-HTTLPR rs25531 genotype on its own already drives the response quite considerably with aripiprazole. In contrast for clozapine, subjects with */LL/* have responses in a range of 2.1 points while for */*/A1A1 carriers the range for PANSS Total is only 1 point. As a general rule, the more a particular genotype drives the response of an antipsychotic the smaller the impact of gene-gene interactions with the other genotypes.

The biggest difference was observed for the */*/D2DRTaq1A1 allele (averaged over the other two genotypes), with the A2A2 genotype favoring the clinical outcome with a difference between 0.3 and 1.2 points on the PANSS Total compared to the A1A1 genotype with the biggest effects on clozapine and risperidone. With regard to the slope of the dose-response, subjects with */*/A2A2 have a greater effect on clinical response. Interestingly, the A2A2 genotype favors drugs with strong D_2_ antagonism (risperidone, haloperidol, olanzapine and paliperidone), while the effect is opposite and much smaller in drugs with weak D_2_R antagonism (clozapine and quetiapine). Aripiprazole is an exception in that the A2A2 genotype also improves clinical response.

The 5-HTT LPR rs25531 genotype has no major impact on clinical response, except for aripiprazole where a substantial better response is achieved in */LL/* carriers (a difference of 2 points on PANSS Total) over the */ss/* carriers with an intermediate response for the */Ls/* carriers, except for paliperidone and risperidone, where the Ls had the best outcome.

The COMT genotype does not affect the clinical outcome in a substantial way for none of the 7 antipsychotics. For instance, the effects are all within a 0.3 point range, with no obvious gene dosage effect. The biggest effect is observed with clozapine in which MV/*/* carriers are 0.42 points better than VV/*/* carriers.

Note the similarity between Paliperidone and risperidone, as the former is the major metabolite of the latter.

### Extra-Pyramidal Symptoms

**FIGURE 3:**
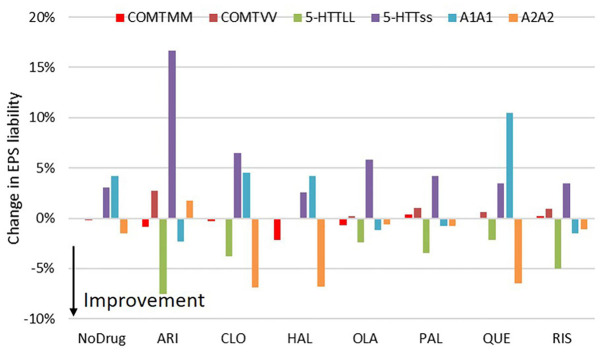
Effect of COMT, 5-HTTLPR rs25531 and D2DRTaq1 genotypes on the EPS liability for the different antipsychotics. The figure shows the changes in probability for patients needing anticholinergic mediation to address their motor side-effects (for instance MM/*/* and VV/*/*) vs. the heterozygote subjects (for instance MV/*/*). All results are averaged over the 9 possible combinations of the other two genotypes with the appropriate distribution depending upon the frequency of the genotypes. While the COMT genotype has no effect on EPS liability, both the 5-HTTLPR and DRD2Taq1 alleles affect this side-effect substantially. The */LL/* genotype, by virtue of its lower 5-HT tone, acts as a 5-HT_2A_ antagonism mechanism; while the */*/A2A2 genotype has a higher dopamine D_2_R expression which effectively acts as less D_2_ antagonism.

We simulated the impact of genotypes on EPS liability for the 7 antipsychotics only at the clinically relevant dose and focused on a clinically calibrated and relevant readout like the fraction of patients prescribed anticholinergic medication as a consequence of their antipsychotic therapy. The simulations (Figure 3) show a substantial variability over the different genotypes with the greatest range observed in aripiprazole (range 14–49% of patients showing motor side-effects) and the smallest in paliperidone (17–28%), olanzapine (24–36%), and quetiapine (17–30%). Note that the placebo subjects already have a range of 16–32% over all the different genotype combinations.

The most important genotype was the D2DRTaq1A allele, with A1 carriers having larger EPS side-effects. The absolute values are highest in drugs with strong D_2_R antagonism (haloperidol, risperidone and paliperidone); however the protective effect of the A2 allele was greater in relative terms for drugs with weak D_2_R antagonism that start already from a low EPS baseline (clozapine and quetiapine side-effects were almost reduced by half).

The 5-HTTLPR rs25531 genotype also affects the EPS liability; */ss/* carriers have a higher probability with */LL/* carriers having a lower probability of side-effects. Aripiprazole is the most sensitive of the antipsychotics; in patients on aripiprazole, */ss/* carriers have a 16% higher chance while */LL/* carriers have a 6% lower probability to show EPS symptoms.

Finally, the effect of the COMT genotype on EPS side-effects is relatively limited (less than 1%).

### Cognitive Impairment in Schizophrenia

Finally we studied the impact of genotypes on a readout for working memory in the presence of antipsychotics using a computational model of a cortical circuit calibrated using the 2-back working memory test (Geerts et al., 2013). Here we only studied the effect of the COMT and 5-HTTLPR rs25531 genotype as we assumed that the D2DRTaq1A1 allele only has an effect on D_2_R availability in the striatum which is not part of the cortical circuit.

**FIGURE 4:**
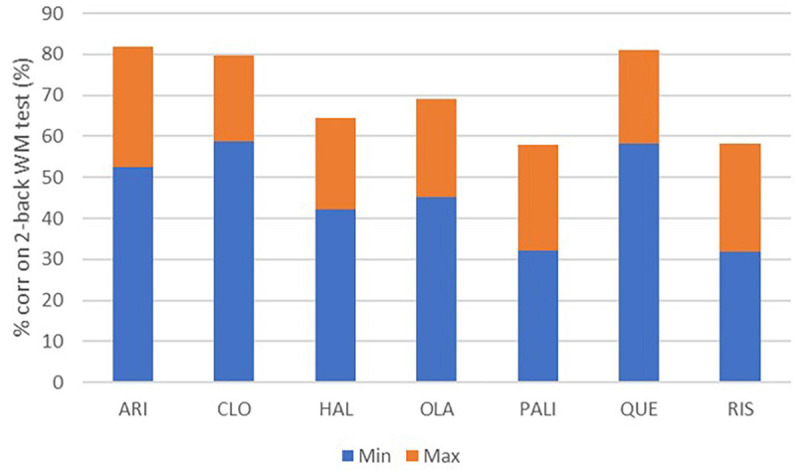
Minimum and maximum effect of cognitive outcome on the 2-back working memory test for the different antipsychotics at the clinically most relevant dose and modulated by 9 different combinations of COMTVal158Met and 5-HTTLPR L/s genotypes. The variability can be substantial (>25 percentage points, a quart of the total dynamical range of the scale) for risperidone, paliperidone and aripiprazole.

Figure 4 illustrates that the range of outcomes between the VV/ss genotype (minimal) and the MM/LL (maximal performance) is considerable, between 22 (for Haloperidol) and 30% (for aripiprazole) on the percentage of accurate responses. In five out of the nine combinations with the two genotypes, clozapine has the best performance, followed by aripiprazole. Conversely, risperidone and haloperidol have the worst outcomes for cognitive readouts. For instance, for a MM/LL subject, the cognitive readout can range from 51 (when on haloperidol) vs. 81% (when on aripiprazole) on the 2-back working memory accuracy task. This can have important consequences for any pro-cognitive therapeutic intervention as the room for improvement is bigger with haloperidol and risperidone.

**FIGURE 5:**
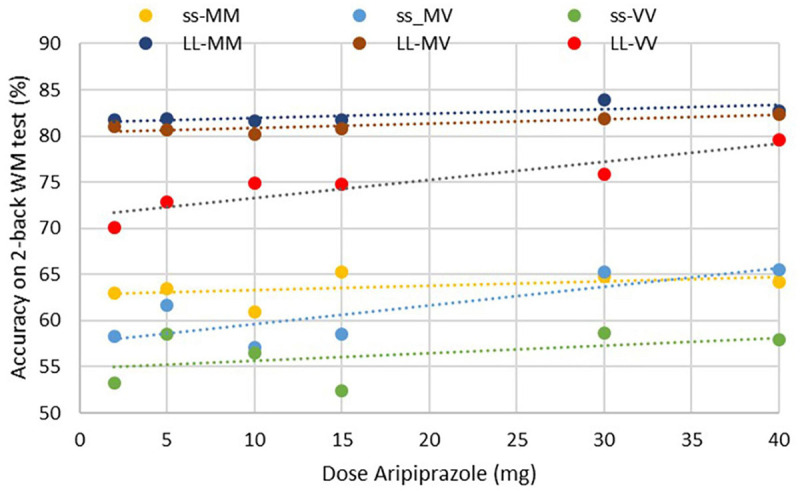
Effect of COMT and 5-HTTLPR rs25531 genotypes on aripiprazole dose-response on simulated cognitive outcome for schizophrenia patients. Most COMTMM-5HTT patients have an essentially flat dose-response and performs quite well; in contrast for COMT VV subjects and to a lesser extent for COMTMV subjects, higher aripiprazole doses significantly improve cognitive outcome.

Furthermore, the genotypes can affect the dose-response of cognitive outcome for each antipsychotic. For example, with aripiprazole the slope of the dose-response in a COMT MV subject is almost 4-fold greater than in a subject with the COMT MM genotype, irrespective of the 5-HTTLPR rs25531 genotype (Figure 5), leading to a difference of 8% in correct responses. Even when averaged over all the genotypes, the slope of cognitive improvement is about 0.12%/mg of aripiprazole, which results in a more than 4 point difference between the doses of 3 and 40 mg. In contrast, there is almost no dose-dependence in cognitive outcome for treatment with most of the other antipsychotics.

With regard to a single genotype, the cognitive outcome is best for subjects with the COMTMM/*/* and the */5-HTTLPR LL/* genotype when compared to the COMTVV/*/* and */5-HTTLPR ss/* genotype respectively.

Table 3 provides an overview of the interaction of the different genotypes with each of the antipsychotics for the three different clinical readouts.

**TABLE 3:** Overview of the sensitivities of clinical outcomes with individual antipsychotics at most relevant clinical dose to single genotypes in patients with schizophrenia.

Drug	Effect on PANSS Total	Effect on EPS	Effect on Cognition
Aripiprazole	Sensitive to 5-HTTLPR and D2DR Taq1A1	Sensitive to D2DR Taq1A1	Modest variability; dose effect; sensitive to COMT/5HTTLPR
Clozapine	Sensitive to D2DR Taq1A1	Sensitive to 5-HTTLPR	Modest variability; sensitive to 5HTTLPR
Haloperidol	Sensitive to D2DR Taq1A1	Sensitive to D2DR Taq1A1	Modest variability; sensitive to 5HTTLPR
Olanzapine	Sensitive to D2DR Taq1A1	Sensitive to D2DR Taq1A1	Modest variability; dose effect; sensitive to 5HTTLPR
Paliperidone	Sensitive to D2DR Taq1A1 and 5-HTTLPR	Limited effect of 5-HTTLPR	Large variability; sensitive to COMT/5HTTLPR
Quetiapine	No effect of genotypes	Sensitive to D2DR Taq1A1	Modest variability; sensitive to COMT/5HTTLPR
Risperidone	Sensitive to 5-HTTLPR and D2DR Taq1A1	Limited effect of 5-HTTLPR	Large variability; sensitive to COMT/5HTTLPR

### Personalized Medicine

Another interesting application of the QSP model involves the identification of the antipsychotic that is best suited for a specific genotype combination, i.e., optimizing the balance for clinical efficacy vs. side-effects based on a specific genotype combination. This is performed by first calculating the weighted average for the different antipsychotics and the three clinical outcomes (i.e., the case of no genotype information). For each individual genotype we then calculated the relative improvement or worsening relative to that average outcome and then rank ordered each antipsychotic (1–7) with lower rank corresponding to a better clinical response or lower side-effect. We then added the rank orders for each of the three outcomes so that we could then identify the antipsychotic with the lowest rank, i.e., the drug that would be suited best.

Table 4 shows the outcome for the 27 different genotype combinations. It turns out that aripiprazole is the best drug for 11 genotypes, followed by clozapine (6) (tied with aripiprazole for two genotypes) and risperidone (6) (tied with aripiprazole for one genotype), olanzapine (3), haloperidol (2), and paliperidone (1) and quetiapine (1).

**TABLE 4:** Selection of the optimal antipsychotic for a given genotype configuration.

COMT	LPR	D2Taq	Frequency	ARI	CLO	HAL	OLA	PAL	QUE	RIS
MM	LL	A1A1	1.56%	3	10	16	16	14	13	12
MM	LL	A1A2	3.13%	4	12	16	13	15	10	14
MM	LL	A2A2	1.56%	5	12	12	15	13	11	16
MM	Ls	A1A1	3.13%	10	8	17	10	14	15	10
MM	Ls	A1A2	6.25%	5	11	15	13	17	9	14
MM	Ls	A2A2	3.13%	11	9	11	17	14	10	12
MM	ss	A1A1	1.56%	17	12	11	8	11	15	10
MM	ss	A1A2	3.13%	17	18	6	12	9	13	9
MM	ss	A2A2	1.56%	18	15	7	10	12	11	11
MV	LL	A1A1	3.13%	5	14	14	15	13	14	9
MV	LL	A1A2	6.25%	6	14	13	18	13	9	11
MV	LL	A2A2	3.13%	10	14	11	18	11	10	10
MV	Ls	A1A1	6.25%	9	10	15	12	11	14	13
MV	Ls	A1A2	12.50%	7	7	14	14	16	11	15
MV	Ls	A2A2	6.25%	10	9	10	19	12	14	10
MV	ss	A1A1	3.13%	16	15	17	14	7	10	5
MV	ss	A1A2	6.25%	16	16	14	10	8	13	7
MV	ss	A2A2	3.13%	16	13	13	13	10	13	6
VV	LL	A1A1	1.56%	7	10	14	10	19	10	14
VV	LL	A1A2	3.13%	8	8	13	11	17	9	18
VV	LL	A2A2	1.56%	10	10	13	8	15	10	17
VV	Ls	A1A1	3.13%	13	11	14	16	8	14	11
VV	Ls	A1A2	6.25%	12	4	14	11	14	12	18
VV	Ls	A2A2	3.13%	12	9	8	18	11	15	11
VV	ss	A1A1	1.56%	16	14	14	4	11	18	7
VV	ss	A1A2	3.13%	17	17	9	9	9	15	8
VV	ss	A2A2	1.56%	18	13	10	11	11	14	7

*For each antipsychotic, individual genotypes are rank ordered for their effects on clinical outcome for efficacy and side-effects. After adding up these rank orders for the three outcomes, one can identify the antipsychotic for each genotype with the lowest rank order and therefore the best overall clinical response. These are shown in yellow. Aripiprazole is the medication that is best for the largest number of genotypes (10) but tied with clozapine for the MV/Ls/A1A2 (the largest group of subjects) and VV/ss/A1A2 combinations. Clozapine and risperidone each are the best choice for six genotypes, Haloperidol and olanzapine for 3 genotypes and quetiapine and paliperidone for one genotype.*

As expected, because the differential effect of the antipsychotics was normalized to the weighted average over genotype, the outcomes were broadly distributed, i.e., there were as many positive as negative changes. The size of these changes, however were different because of the pharmacological properties of the antipsychotics and provided a rationale for personalized antipsychotic selection.

## Discussion

This report documents the anticipated effects of three common genotype variants, i.e., COMTVal158Met, 5-HTTLPR rs25531, and D2DRTaq1A1 on PANSS Total clinical response and EPS side-effects and cognitive impairment.

Because of the rich pharmacology, the pharmacodynamic interactions between the antipsychotics and the genotypes are very complex, leading to a unique and antipsychotic-specific outcome for each of the 27 different genotypes configurations. Ideally these predictions would need to be corroborated with experimental clinical findings to be of value. Unfortunately, there are not many well-designed clinical studies for testing these observations. In clinical studies on single genotypes, the other genotypes are not always available which makes it difficult to test the outcomes of this QSP analysis. Given the distribution of the genotypes, where the triple homozygotes account for 1/64, population studies need to account for at least a few thousand subjects with full information not only on the genotypes but also on the dose of the antipsychotic and possibly other comedications. Many clinical studies report on the impact of a single genotype on the clinical outcome. In our platform, these single-gene patient populations can be simulated by averaging over the two other genotypes with the appropriate weighting factors. As demonstrated in Tables 1, 2 and explained in detail below, the platform outcomes are grossly in line with some of the anecdotical study reports.

The simulations suggest that the DRD2Taq1 A2A2 carriers with a higher striatal D_2_R expression have a better clinical outcome when treated with strong D_2_R antagonism; this is likely due to the placebo response that tends to be lower in these carriers. In contrast, drugs with weaker D_2_R antagonism are not able to overcome this higher baseline of D_2_R expression and activation by the hyperdopaminergic tone. In our simulations, when averaged over the two other genotypes, the DRD2 had modest effects on the outcome of olanzapine (within 1–1.5 points on PANSS Total), much less than aripiprazole and risperidone on PANSS Total and aripiprazole and quetiapine on EPS side-effects. Overall, the effect size (a few points at most on PANSS Total) is small and unlikely to be detectable.

With regard to EPS however, A2A2 carriers tend to be more protected due to the higher D_2_R levels in the placebo in haloperidol, clozapine and quetiapine cases compared to the heterozygote subjects, while the A1A1 genotype exacerbates the outcomes. The effect of this genotype on EPS liability with other antipsychotics is more limited. Clinical studies indeed suggest that the DRD2 genotype does not affect the adverse EPS effects of ziprasidone, olanzapine and perazine (Tybura et al., 2014).

We further assumed that this genotype did not affect the D_2_R availability in cortical areas – which is already much lower than the striatal expression level (Seaman et al., 2019) and therefore did not affect cognitive readout. However, there might still be a small effect on expression level that was below the detection limit of the imaging study or striatal dopamine might affect cognitive performance, as a recent study suggest (Veselinovic et al., 2018).

The effect of the 5-HTTLPR genotype in schizophrenia has not been studied in the clinical setting except for one study suggesting an association between the 5-HTTLPR genotype and the risk for schizophrenia in a South Indian population (Vijayan et al., 2009), but not in a Japanese population (Ikeda et al., 2006).

Our simulations suggest that the LL-carriers do have a lower EPS side-effect liability for most antipsychotics which is driven by the lower 5-HT tone in cortical areas. This reduces 5-HT_2__*A*_ activation which furthers amplifies any 5-HT_2__*A*_ antagonism that atypical antipsychotics might have and for example turns haloperidol’s profile into a relative atypical antipsychotic. However recent studies suggest the presence of a tri-allelic impact with an additional G-A mutation in the L-form of the promotor (Hu et al., 2006) with the A-form, but not the G-form enhancing the L-phenotype on 5-HTT transporter expression.

In patients with major depression, response to antidepressants is strongly modulated by the 5-HTTLPR rs25531 genotype (Staeker et al., 2014). As many atypical antipsychotics have a complex serotonergic pharmacology, it makes sense that this genotype also affects clinical response in schizophrenia.

Interestingly, subjects with the 5-HTTLPR L/L genotype, who have lower basal serotonin levels perform better on cognitive readouts for all antipsychotics. This is likely due to the lower 5-HT_3_ and 5-HT_6_ activation levels that improve neuronal firing and network stability. A meta-analysis of ondansetron studies in schizophrenia suggest a beneficial effect on PANSS Total, PANSS negative and general psychopathology scales and a reduction on extrapyramidal symptoms, but somewhat mixed results on cognition (Zheng et al., 2019). On the other hand 5-HT_6_ antagonism has been shown to improve cognition in preclinical models (de Jong and Mork, 2017) but with mixed results in clinical Alzheimer’s trials (Maher-Edwards et al., 2010). Also, the 5-HTTLPR L/L genotype is over-represented in Obsessive-Compulsive disorder (Hu et al., 2006) and in aggression associated with Alzheimer’s disease (Sukonick et al., 2001). We speculate that this could be due to the fact that the 5-HTTLPR L/L allele overstabilizes representations in the cortical network at the expense of flexibility (Mekern et al., 2019).

When studying a single genotype for cognitive outcome, the COMT Met158Met subjects are responding better than COMT Val158Val carriers, in line with a meta-analysis (Huang et al., 2016). In this study the authors showed that the effect of the genotype was limited to atypical antipsychotics. The NESSy trial study (Veselinovic et al., 2019) suggest that first-generation antipsychotics have a worse performance – in line with our predictions on haloperidol. The majority of individual trials from Table 2 also support the conclusion that the COMT Met genotype has a greater cognitive benefit. The platform suggest that this is likely through a direct D_1_-mediated effect on voltage-gated ion channels and a D_4_-mediated effect on AMPA receptors that modulates excitability of pyramidal neurons. However there are notable exceptions, suggesting that the relation between COMT genotype and cognitive performance is modulated by other factors, such as the inverse U-shape dose-response of dopaminergic effects on cognition (Chou et al., 2013).

Interestingly, a small study suggested a relationship between higher dopamine occupancy and cognitive performance on the n-back working memory test for aripiprazole in patients with schizophrenia (Shin et al., 2018). When appropriately averaged over all the genotypes. our model predicts a dose-dependent improvement with average slope of 0.12% higher accuracy/mg aripiprazole in the 2-back working memory test, probably due to greater 5-HT_1__*A*_ agonism.

Gene-gene interactions have been observed in clinical studies, such as between the COMT and DRD4 genes on the response to clozapine (Rajagopal et al., 2018).

The QSP platform in principle allows to identify the drug with the best benefit of clinical efficacy (PANSS Total) over side-effects (motor symptoms and cognitive deficit) for each individual genotype configuration. Aripiprazole and clozapine score best for the highest number of genotypes, including the heterozygous combinations. Such an approach could be useful for personalized medicine, where treatment could be started by the best antipsychotic for the patient specific genotype configuration.

Importantly, the simulations suggest that the choice of the antipsychotic not only affects the baseline of clinical readout, but also can affect the dose-response of an augmentation drug in clinical trials for Cognitive Impairment associated with schizophrenia (CIAS). Although not presented here, the multiple pharmacodynamic interactions between an antipsychotic and genotypes might well effect differentially the impact of a novel drug target that affects the excitatory-inhibitory balance in the cortex and/or other modulating neurotransmitter systems. Our approach offers a tool to identify possible negative pharmacodynamic interactions that can be mitigated in a clinical trial design.

Overall, the anticipated effect size of the different genotypes on PANSS Total is small and likely undetectable, however, there are considerable differences on EPS side-effects and cognitive performance.

We acknowledge that in clinical practice, other clinical phenotypes such as metabolic dysfunction and negative symptoms are important; however they are beyond the scope of this project.

A major limitation of this approach is that the physiological effect of the genotypes needs to be well documented, usually based on human imaging studies. The physiological effect of most genotype variants is not known, although, in principle, studies using neuronally differentiated hIPSC could in principle provide additional insights. This paper is a proof-of-concept for simulation of the pharmacodynamic interactions between well- defined genotypes and antipsychotic treatment and can be extended to other genotypes once sufficient information will become available. Furthermore, we focus here only on the pharmacodynamic interactions between genotypes and a single antipsychotic; in clinical practice however, patients are often on a combination of comedications with CNS active properties. In principle, however, this mechanism-based platform is able to simulate the pharmacodynamic interactions between comedications based on their pharmacological properties; as has been applied to a blinded predictive study of motor side-effects as a consequence of two antipsychotics in a clinical practice sample of schizophrenia patients (Kadra et al., 2018).

Another issue is the determination of the effect of genotypes on neurotransmitter dynamics; for instance although the PET radiotracer NNC-112 originally has been identified as a selective D_1_ antagonist (Andersen et al., 1992), *in vivo* in the baboon there is only a sixfold to fourteenfold selectivity over 5-HT_2__*A*_ (Ekelund et al., 2007). Although there is little evidence that the serotonergic component has a great impact on cortical dopamine dynamics, in principle this could been addressed with a sensitivity analysis around the postulated half-lives. In addition, for many other genotypes we still don’t know the physiological consequences so they cannot be implemented in the QSP platform.

Similarly the effect of smoking can be implemented through modulation of nicotinic AChR resulting in complex non-linear interactions for cognitive impairment (Geerts et al., 2015).

Another limitation is the implementation of the schizophrenia changes. Although the model includes four aspects of the changes documented in patients (striatal hyperdopaminergic and cortical hypo-dopaminergic state, cortical Glutamate and GABA dysfunction and increased noise), there are probably changes in other neurotransmitter systems or neuronal pathways. The effect of these putative extensions on the clinical responses of the antipsychotics is unknown.

The model is not intended to simulate the effect of metabolism genotypes such as CYP450 enzymes that drive drug exposure and likely clinical outcome. This type of interactions has been the subject of intense study [for example see (Kneller et al., 2020)]. Finally, the model predicts the pharmacodynamic interaction between functional processes (here identified by the specific genotypes) and the pharmacology of individual antipsychotics. It is certainly possible that other genotypes acting in the same circuit or pathway can be found that affect the clinical outcome in the same way. In this regard, the platform is merely a hypothesis-generating engine to identify key pathways interacting with various antipsychotics.

In contrast to this knowledge-driven QSP approach, Big Data based analytics derive insights from large datasets, usually from electronic health records that can provide correlations between clinical efficacy and/or side-effects and specific genotypes. However as discussed above, to capture all possible combinations, including the effect of antipsychotic dose, smoking status and other comedications, one needs high quality datasets of many thousands of subjects. In the absence of such high-quality datasets, mechanism-based modeling approaches based on domain expertise and sound pharmacological principles could be a valuable alternative.

In summary, this paper presents a new computer-based approach to quantify the pharmacodynamic consequences of gene-gene interactions on the clinical outcome of antipsychotics in schizophrenia, based on neuropharmacology and neurophysiology domain expertise. Further validation of this approach awaits better clinical datasets.

## Data Availability Statement

The original contributions presented in the study are included in the article/Supplementary Material, further inquiries can be directed to the corresponding author/s.

## Author Contributions

AS developed the QSP model. HG conceived the experiment, performed the simulations, and wrote the manuscript. Both authors contributed to the article and approved the submitted version.

## Conflict of Interest

AS and HG were employed by company In Silico Biosciences. HG was employed by company Certara QSP.

## Publisher’s Note

All claims expressed in this article are solely those of the authors and do not necessarily represent those of their affiliated organizations, or those of the publisher, the editors and the reviewers. Any product that may be evaluated in this article, or claim that may be made by its manufacturer, is not guaranteed or endorsed by the publisher.
