# Supplementary material for: Toward Predicting Impact of Common Genetic Variants on Schizophrenia Clinical Responses With Antipsychotics: A Quantitative System Pharmacology Study
Source: Front Neurosci. 2021 Sep 29;15:738903. doi: 10.3389/fnins.2021.738903 (PMC8511786; doi:10.3389/fnins.2021.738903)
Supplement: Supplementary file 1 [file Table_1.DOCX]

Supplement to

THE IMPACT OF COMMON GENETIC VARIANTS ON SCHIZOPHRENIA CLINICAL RESPONSES WITH ANTIPSYCHOTICS. A QUANTITATIVE SYSTEM PHARMACOLOGY STUDY

Hugo Geerts ^1 ,2^ , Athan Spiros ^1^

^1^ In Silico Biosciences, Berwyn , Pennsylvania, ^2^ currently at Certara-SimCyp

METHODS.

1. The Receptor Competition Model

An important issue for any modeling of clinical situations is the target engagement of the antipsychotics at their respective clinical dose. To calculate the functional free concentration of the drug, we use the receptor competition model, a set of ordinary differential equations that describe the competition between neurotransmitter, drug, its metabolite and a possible radiotracer [[1](#_ENREF_1)].

Basically this is a set of ordinary differential equations that describes the time-dependent changes in pre- and postsynaptic receptor activations, neurotransmitter and drug levels in the synaptic cleft and amount of binding to different receptors under realistic presynaptic firing conditions of the endogenous neurotransmitter. The following differential equations simulate the binding of up to four agents at pre- and postsynaptic receptors based upon their respective affinities [[1](#_ENREF_1)].

##### $\partial[R_{n}]/\partial t=k_{on}^{n}\times[NT]\times[R_{f}]-k_{on}^{n}\times K_{d}^{n}\times[R_{n}]$ Eq S1

Where n is for neurotransmitter. Similar equations are used for drug1, drug2 and tracer. R_f_ denotes the level of free receptor; k_on_ is the on-rate (usually diffusional controlled if not experimentally determined) and K_d_ is the dissociation constant.

Exponential decay of the neurotransmitter is defined as

[NT](t) = [NT(0)]*exp(-t ln(2)/halflife) (Eq S2)

where halflife is the half-life of the decay process. This is modulated by transporters (in the case of DA, 5-HT and NE) or enzymes (Acetylcholinesterase for Ach and Catechol-O-Methyl transferase for DA and NE) and will be dependent upon the COMTVal158Met and 5-HTTLPRS rs23351 genotype.

All differential equations are solved with a fourth-order Runge-Kutta method with a time step of 0.01 msec.

# In addition, the release can be modulated by a depression or facilitation mechanism [[2](#_ENREF_2)]. Instead of using internal Ca levels to determine dopamine release, we consider the facilitation and depression of dopamine release based solely on the amount of time elapsed since the previous firing using a phenomenological equation. Thus, the amount of dopamine released is based both on the history of firing and the activation level of the presynaptic D2 autoreceptors. If we denote the time of the n^th^ firing by t_n_, then the release amount is modified based on all previous firings as follows

$release_{new}=release\left(1+\sum_{i=1}^{n-1} w_{f}\exp\left[-k_{f}(t_{n}-t_{i})\right]-w_{d}\exp\left[-k_{d}(t_{n}-t_{i})\right]\right)$ Eq S4

where w_f_ is the facilitation weight, w_d_ is the depression weight, k_f_ is the decay rate of facilitation and k_d_ is the decay rate of depression.

All the parameters that describe the presynaptic neurotransmitter physiology are calibrated with preclinical experiments using rapid-cyclic fast voltammetry on levels of neurotransmitters [[3](#_ENREF_3), [4](#_ENREF_4)].


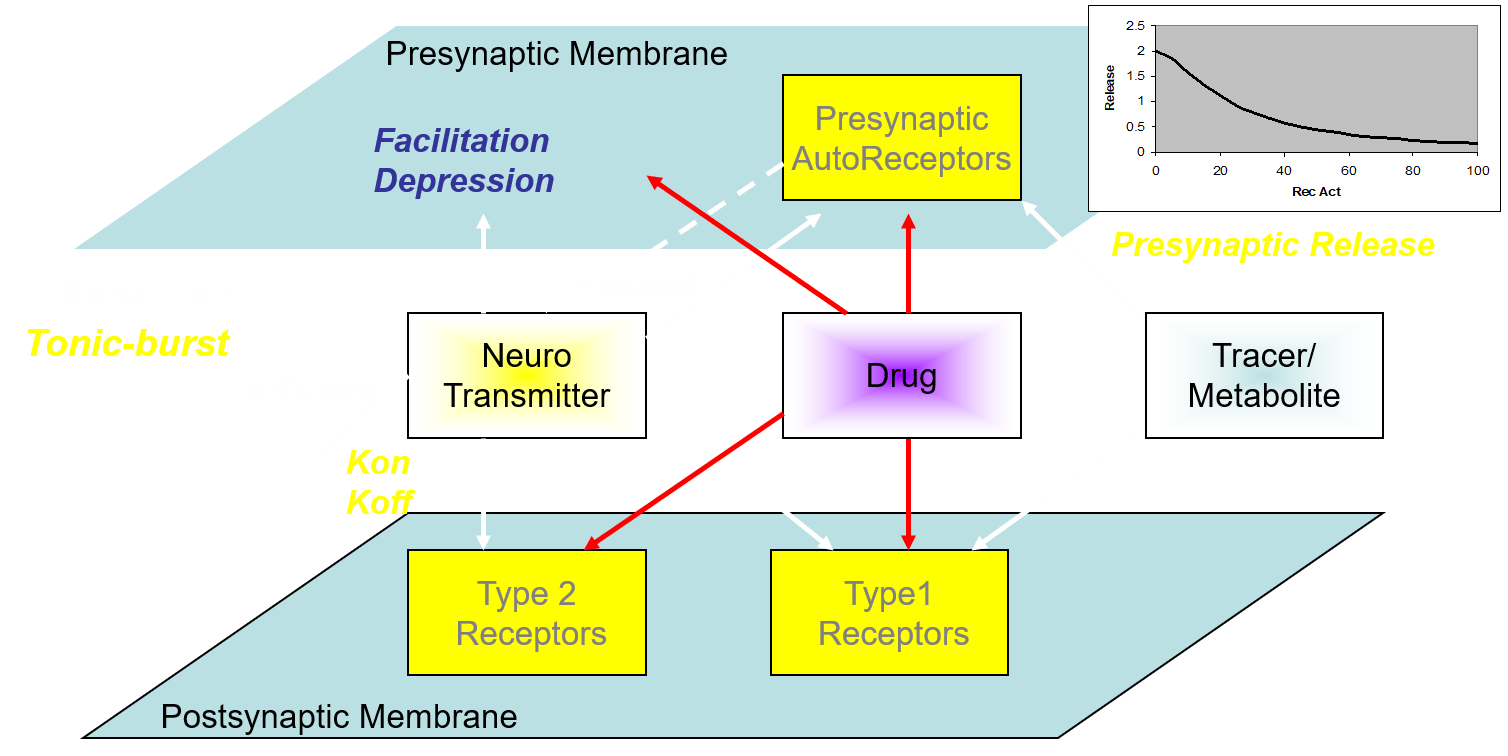


Fig 1. Representation of the Receptor Competition Model as described in the text.

1. Applications of the Receptor Competition Model.

2A Determining the functional intrasynaptic concentration of the antipsychotics active moiety

The functional free intrasynaptic concentration of the drugs is determined by simulating the competition between total active moiety (including the active metabolite) and tracer at the postsynaptic D_2_R in a PET radiotracer displacement study [[5](#_ENREF_5)]. For cortical drug concentrations we use tracer displacement data of various antipsychotics on FLB417 [[6](#_ENREF_6)]. Using the competition model between neurotransmitter, drug and tracer for binding at the postsynaptic receptor, we can determine the drug concentration that corresponds to a clinically measured radiotracer displacement. This value for the drug concentration is the free and functional intra-synaptic concentration that is dependent upon the PK properties of the drug and is used in further calculations. For all the clinical studies we assume steady-state plasma and target engagement values.

We can then use this free functional concentration of the active moiety to calculate the postsynaptic receptor activation in other non-dopaminergic D_2_ synapses, dependent upon the affinity of the drug, its metabolite and the endogenous neurotransmitter (such as 5-HT or Ach). The change in the appropriate postsynaptic receptor activation is then used to derive the change in voltage-mediated ion channels that drive the excitability of the network (see below).

Because raclopride displacement is measured functionally it takes into account many confounding issues such as blood-brain barrier transport and free fraction etc. and reflects the actual true concentration of the drug.

2B. Implementation of the effect of COMTVal158Met and 5-HTTLPR rs23351 genotype effects

We use the same model, but now in the absence of any drugs to simulate the observed displacement of the D1 radiotracer NNC-112 as a function of the genotype in unmedicated healthy volunteers [[7](#_ENREF_7)]. The COMT genotype affects the stability of this enzyme that breaks down dopamine and norepinephrine and therefore the level of dopamine that competes with the radiotracer for binding on the postsynaptic D1 receptor. A change in cortical dopamine half-life from 100 msec in the COMT VV to 130 msec for COMTMV and 160 msec for the COMTMM genotype fits the observed changes in the binding potential. Similarly, the 5-HTTLPR rs23351 mediates the expression of the 5-HT transporter to the extent that experimentally observed binding potential of the 5-HT4 specific radiotracer SB210 in unmedicated healthy volunteers can be fitted by a half-life of 50 msec for the ss, 75 msec for the Ls and 100 msec for the LL genotype.

1. **Cortical-striatal-thalamocortical circuitry model for PANSS Total.**

Network Architecture. Fig 1 shows the striatum, STN-GP circuitry, and thalamo-cortical circuitry implementations of the QSP model, based on previous implementations [[8-10](#_ENREF_8)] where we have added relevant neuromodulator receptor effects (see Supplementary Table 2) and subsequently implemented a schizophrenia pathology based on human imaging studies. The model is based on documented projections in non-human primates and human brain and represents the associative loop.

Connectivity. Projections from pyramidal cells with cell soma in layer IV and V drive activity of two types of medium spiny neurons that drive direct and indirect pathway respectively. The direct pathway (globus pallidus interna) then connects to the thalamus; the indirect pathway connects to the globus pallidus externa and the subthalamic nucleus and couples back to the globus pallidus interna. The thalamus projects back to the cortex, closing the loop. In addition, the cortex affects the subthalamic nucleus through a hyperdirect pathway.

Neuronal Model. Each neuronal cell type is modeled with biophysically realistic membrane conductances to simulate their functional role in the circuit; changes in receptor activation as determined by the receptor competition model (see above) caused by the pharmacology or the pathological state can change the spiking activity. Each compartment of the model neuron follows the membrane current balance equation of the Hodgkin-Huxley formalism [[11](#_ENREF_11)]. The membrane potential, V, is then computed by numerically integrating the equation for each compartment CdV/dt=Σ g_a_(V-E_a_)+I_ex_, where C is the membrane capacitance, g_a_ is the ionic conductance of a specific type ion channel, and E_a_ is the reversal potential of that ion channel that is then summed over all types of ion channels, a, in each model compartment, and I_ex_ represents an externally applied current from synaptic currents.

Striatum component. The striatum model simulates the processing capacity of medium spiny neurons (MSN) in the ventral striatum or nucleus accumbens [[12](#_ENREF_12)] and calculates the excitability of the two types of MSNs; D_1_ cells that project to the direct pathway and D_2_ cells projecting to the indirect pathway; both are driven by afferent cortical projections.

In the 10 MSN cells of the direct pathway, D_1_ receptors affect the K_ir_ channel [[13](#_ENREF_13)] while in the 10 MSN cells of the indirect pathway, D_2_ receptor activation mainly affects the A-type K^+^ current. For both types of MSN, D_2_ receptor activity modulates the presynaptic Glu release on the afferent cortical fibers that drive the excitability of the MSN neurons.

We elaborate on the modeling of the D1R+ MSN neuron as an example

## The MSN in the direct pathway are directly modulated by D_1_R through the Kir2 current [[13](#_ENREF_13)]. The time-dependent D_1_ activation is described with a different function u (t) for both weak (tonic) and strong (burst) stimulating pyramidal signals. The function u is determined by dopamine neurotransmitter release, postsynaptic D_1_R activation and intracellular pathways activation. Because these processes are not known in great detail, we describe this dopamine activity by means of a phenomenological function u(t) [[14](#_ENREF_14)]. Tonic firing is described by a relatively slow increase to u_max (rate ~ 25 ms), a 5msec duration and a slow decrease (rate ~ 50 ms), while burst firing is described by a rapid increase to u_max (rate ~ 1 ms), a long duration at this maximum (~100 msec) and a slow decay (rate ~ 100 ms) [[14](#_ENREF_14)]. The parameter u_max is determined from the average D_1_R activation in the receptor competition model.

##### This dopaminergic signal begins 1250 ms into the simulation and continues for 19 seconds.

##### The inward rectifying potassium current, Kir2, is modified by the dopamine D_1_R activation u [[13](#_ENREF_13), [15](#_ENREF_15)] so that the total current, $I=u\cdot I_{Kir2}$. With a conductance, g_K_, and a reversal potential, E_K_ = -90 mV, the current takes on the form $I_{Kir2}=g_{K}\left(V-E_{K}\right)$ with a voltage dependent form $g_{K}=\bar{g}_{K}\frac{1}{1+\exp\left(-\frac{V-V_{h}^{K}}{V_{c}^{K}}\right)}$ Eq S5

##### where $\bar{g}_{K}$ = 1.2 mS/cm^2^ is the maximum conductance, V_h_ = -111 mV is the value of the membrane potential that causes half activation and V_c_ = -11 mV describes the sensitivity of the change ([[14](#_ENREF_14), [16](#_ENREF_16)].

##### The dopamine signal u(t) is then introduced with a 4Hz firing pattern as defined above with a maximum u_max given by

##### u_max = 1 + d1Adj*(d1Tonic/15.84). Eq S6

##### For the burst case, u_max is determined by the function

##### u_max = 1 + 2d1Adj*(d1Burst/31.21) Eq S7

##### d1Tonic and d1Burst are the average D_1_R activation levels as determined from the receptor competition model during a tonic and burst firing frequency respectively. d1Adj is a free parameter that can be adjusted to optimize the correlation between the model outcome for different drug-dose combinations and the clinical reported effects on PANSS total for the same drug-dose combinations (see below). We calculate then the change in the membrane potential (V) of the GABA spiny neuron due to the Kir2 current as $-C\frac{dV}{dt}=u\cdot I_{Kir2}$ where C=1, V is the membrane potential, I_Kir2_ is the inward rectifying potassium current and u (t) is a measure of the D_1_-R activation as defined above.

D_1_-R activation increases the L-Ca current in the GABA spiny neuron ([[17](#_ENREF_17)] and affects the voltage range of the up state. The passive calcium current, L-Ca, is modeled with the Goldman-Hodgkin-Katz equation with concentrations for internal calcium to 10 pmol/cm^3^ and the external calcium to 2 μmol/cm^3^. The maximum permeability is 4.3 nm/s.

We further calculate the change in the membrane potential (V) of the GABA spiny neuron due to the L-Ca current as $-C\frac{dV}{dt}=u\cdot I_{L-Ca}$ where C=1, V is the membrane potential, I_L-Ca_ is the calcium current and u is a measure of the D_1_-R activation (see above). Furthermore, the calcium current is defined as

$I_{L-Ca}=P_{L-Ca}\left(\frac{4VF^{2}}{RT}\right)\left(\frac{[Ca]_{i}-[Ca]_{o}\exp\left(\frac{-2VF}{RT}\right)}{1-\exp\left(\frac{-2VF}{RT}\right)}\right)$ Eq S8

where F is Faraday’s constant, T is the temperature, R is the gas constant, [Ca]_i_ is the internal and [Ca]_o_ is the external calcium concentration. P_L-Ca_ is the membrane potential which is also voltage dependent as

#### $P_{L-Ca}=\bar{P}_{L-Ca}\frac{1}{1+\exp\left(-\frac{V-V_{h}^{L-Ca}}{V_{c}^{L-Ca}}\right)}$ Eq S9

#### where $\bar{P}_{L-Ca}$ is the maximum permeability and with $\bar{P}_{L-Ca}$ = 4.3 nm/s, V_h_ = -35 mV, V_c_ = 6.1 mV ([[18](#_ENREF_18)].

STN-GP circuitry component. The computer model for the direct and indirect pathways is extended from the Rubin & Terman model [[10](#_ENREF_10)] of STN, GP_i_, and GP_e_ [[8](#_ENREF_8)]. Each nucleus contains 16 neurons with the following membrane currents: sodium current (Na^+^), delayed rectifier potassium current (K_dr_^+^), T-type calcium current (Ca_T_^++^), L-type calcium current (Ca_L_^++^) and a leak current.

The STN, GP_e_, and GP_i_ population is divided in 2 groups of 8 cells each. The D_2_-type MSN cells from the striatum synaptically connects to the GP_e_ neurons and the D_1_-type MSN cells synaptically connect to the GP_i_ neurons both with a GABA current. Each GP_e_ cell receives inhibitory input from 3 other GP_e_ cells in addition to excitatory input from three randomly chosen STN cells while each STN cell receives inhibitory input from 3 GP_e_ cells. Furthermore, each GP_i_ cell receives inhibitory input from 1 GP_e_ neuron and excitatory input from 1 STN neuron. All the TC cells of the thalamus receive inhibitory input from 8 GP_i_ cells.

Thalamo-cortical component. The thalamus model is based on the circuitry and cellular properties of 4 thalamocortical neurons (TC) and 4 reticular neurons (Re). The TC are excitatory, glutamatergic relay neurons that pass sensory information to the cortex model described in [[9](#_ENREF_9)]. The Re are inhibitory, GABAergic feedback neurons that receive inputs from, and inhibit, TC neurons. The synaptic interaction between these neuronal types, and their intrinsic membrane properties, leads to oscillations, suppression and amplification of multiple input signals.

The spiking property of TC neurons is driven by a fast sodium channel, Na^+^, a fast potassium channel, K^+^, a low-threshold Ca^++^ channel, iTC, a hyperpolarization-activated cation channel, Ih, a potassium A channel, Ka, and a potassium leak channel. For an Re cell, a fast sodium channel, Na^+^, a fast potassium channel, K^+^, a low-threshold Ca^++^ channel, iTC , and a potassium leak channel are implemented. All parameters (channel kinetics, current densities, morphology and synaptic strengths) were set at the values given in [[9](#_ENREF_9)].

### The cortex contains pyramidal cells (Pyr) and inhibitory basket cells (BC) and is derived from the QSP model originally described for working memory [[4](#_ENREF_4), [19](#_ENREF_19)]. Basically this version consists of 20 two-compartment pyramidal cells and 10 two-compartment GABA inhibitory neurons. 60% of the interneurons synapse on pyramidal cells, the remaining part forming a recurrent microcircuit. The pyramidal cell model consists of 3 compartments: a soma, an apical proximal dendrite and an apical distal dendrite. Each pyramidal cell receives (1) synaptic input from other pyramidal cells on its apical and basal dendrites, (2) synaptic input from inhibitory interneurons onto its soma, (3) receives excitatory and inhibitory synaptic input from a background noise source that represent other pyramidal cells and inhibitory interneurons that are not explicitly represented in the model and is driven by thalamic projections. Upon firing an action potential, glutamate is released onto postsynaptic receptors (AMPA-R and NMDA-R). The pyramidal cell model includes a number of voltage gated channels that control the membrane currents and state of depolarization: Naf, Nap, Hva, Kdr, Ks, KCa, and Leak. Each of these channels is modeled using a Hodgkin-Huxley framework with density and maximal conductance parameters as in [[20](#_ENREF_20)]. The lengths and diameters of the cylindrical compartments are 6.14 μm and 86.3 μm for the soma and 400 μm and 2.6 μm for both apical proximal and distal dendrite.

A value of 30 k$\Omega$*cm^2^ for the specific membrane resistance *R*_m_, 1.2 mF/cm^2^ for the membrane capacity C_m_, 150 $\Omega$*cm for the cytoplasmatic (axial) resistivity R_i_, [[21](#_ENREF_21), [22](#_ENREF_22)] and a leakage reversal potential of 270 mV is used. These values (together with the active processes) resulted in a resting membrane potential of - 66 mV, a cellular resistance *R*_IN_ of 164 M$\Omega$ and a membrane time constant of 36 ms. For dendritic spines the effective dendritic *C*_m_ was increased and the effective dendritic *R*_m_ was divided by a factor of 1.92 [[23](#_ENREF_23)].

An mGluR-dependent *delayed afterdepolarization* current [[24](#_ENREF_24)] was implemented that can increase the spiking rate of pyramidal cells for several seconds with a current injection shaped as an alpha function initiated at time 2 sec.

## Fast-spiking, basket cell-type interneurons consist of two compartments: a soma and apical dendrite and receives excitatory synaptic input from pyramidal cells onto its dendrites and inhibitory synaptic input from other interneurons onto its soma, in addition to excitatory and inhibitory synaptic input from a background noise source that represent other pyramidal cells and inhibitory interneurons that are not explicitly represented in the model. When the interneuron fires an action potential, the transmitter GABA is released onto postsynaptic receptors (GABA_A_-R). The inhibitory interneurons model includes two voltage gated channels that control the membrane currents and state of depolarization: Na, K. The soma of the interneuron is a cylindrical element with length=15 μm and diameter=15 μm, while the dendrite has a length of 150 μm and a diameter of 10μm. We used a value of 100 k$\Omega$*cm2 for R_m_, 1.0 mF/cm2 for C_m_, 150 $\Omega$*cm for R_i_, and -268 mV for E_leak_.

The pyramidal cells project to the MSN neurons in the striatum for both direct and indirect pathways and to the STN for the hyperdirect pathway. The parameters for the voltage-gated ion-channels and the coupling factors between various GPCR and their effector targets are determined by preclinical data in non-human primates [[25](#_ENREF_25)] and subsequent calibrations [[4](#_ENREF_4), [19](#_ENREF_19)] and predictions of clinical efficacy [[26-28](#_ENREF_26)].

*Synaptic currents.* The synaptic connections are based on the kinetics of AMPA, NMDA, GABA-A, and mGluR currents. Excitatory synapses include both AMPA and NMDA currents with fixed maximal inward depolarizing conductance (g), rise time constant (*t*_rise_), and decay time constant (*t*_decay_) and reversal potential of 0 mV [[29](#_ENREF_29)]. The following equation describes the Na^+^ conductance (g_glu_) of both AMPA and NMDA receptors used in this model (Mg^++^block for NMDAR not shown): g_glu_(t) = g(exp(-t/*t*_decay_) – exp(-t/_trise_). Inhibitory chemical synapses represent GABA-A receptor currents using a similar scheme as excitatory synapses, with the GABA-A (chloride) reversal potential and kinetics associated with each cell type. Each model neuron receives fluctuating currents to simulate background synaptic bombardment by excitatory and inhibitory neurons [[30](#_ENREF_30)].

*Implementation of drug effects on receptor downstream effects.* Here we elaborate on the strategies for linking postsynaptic receptor activation changes by either pathology or drug effect on effector functions such as voltage-gated ion channels. We document the cortical dopamine effects in detail; a similar strategy can be used for other receptor types at other sites in the model.

### Dopamine D_1_R activation is first normalized as D1_eff= (D1_A - D1_actC)/D1_actC, where D1_A and D1_actC are the actual D1R activation levels (with pathology and drug effects) and the healthy control levels. In the cortex, D1R affects Hva channels as g_Hva’= g_Hva * (1 - D1_eff / 9) and the leak channel g_L’=g_L*(1-D1_eff/3) where the denominators illustrate the maximum response (11% for Hva and 33% for the leak channel).

### D_1_R activation increases the NMDA channel conductances g_NMDA’= g_NMDA * (1 – Param_D1_NMDA* D1_eff ), reduces the AMPA channel conductances g_AMPA’= g_AMPA * (1 – Param_D1_AMPA* D1_eff ), and reduces the GABA channel conductances g_GABA’= g_GABA * (1- Param_D1_GABA * D1_eff ) [[31](#_ENREF_31)].

### In all cases we assume a linear relationship between receptor activation and subsequent effects on ion-channels. The effects of D_2_S receptor as presynaptic autoreceptor has been taken into account by the cortical dopamine receptor competition model. Furthermore, D_2_R activation modulates AMPA conductance with the following formula

g_AMPA’= g_AMPA * (1 – Param_D2_AMPA* D2_eff ), (Eq S10)

where D2_eff = (D2_A-D2_ACont)/D2_ACont.

### Dopamine D_4_R activation is first normalized as D4_eff=(D4_A – D4_actC)/D4_actC, where D4_A and D4_actC are the actual D4R activation levels and the healthy control D4R activation levels. D4R activation increases the AMPA channel conductances g_AMPA’= g_AMPA * (1 – Param_D4_AMPA* D1_eff ). All these cortical parameters have been calibrated using clinical observations in Alzheimer’s Disease [[4](#_ENREF_4)] and cognitive impairment in schizophrenia [[19](#_ENREF_19)] and are therefore fixed in this project.

Calibration of the network is performed using group-average data on changes in PANSS total from 43 drug-dose combinations of Phase III and Phase IV clinical studies with 24 antipsychotics extracted from 147 papers published between 1992 and 2010 [[32](#_ENREF_32)].


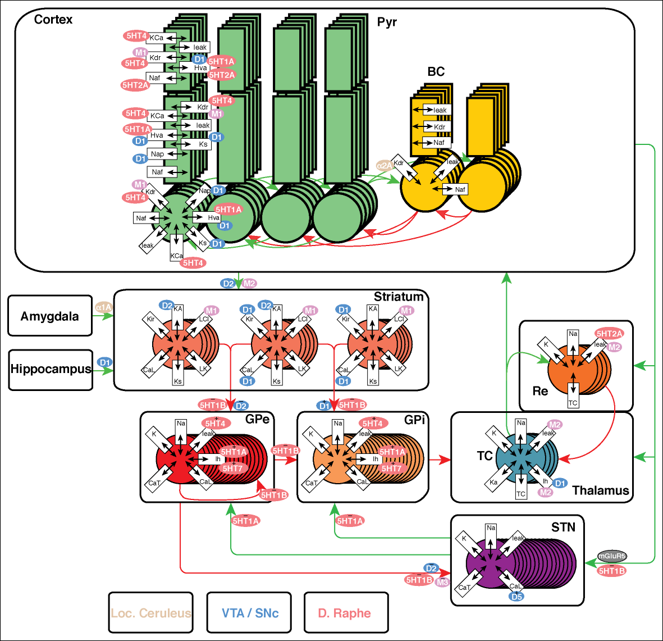


Fig 2. Schematic representation of the cortico-striatal-thalamocortical Quantitative Systems Pharmacology model as described in the text

1. EPS Model

This QSP model is based on the known neuro-anatomical pathways linking Supplemental Motor Area-SMA cortex to different parts of the dorsal striatum, including a striatal part with two types of medium spiny neurons (MSN) : D_1_+ MSN projecting into the Globus Pallidus Interna (GP_i_) of the direct pathway, D_2_+ MSN neurons projecting into the Globus Pallidus Externa (GP_e_) and the subthalamic Nucleus( STN) of the indirect pathway. The subthalamic nucleus projects back into the GP_i_ and the GP_i_ projects the combined activity out to the thalamus. A Thalamic Reticular Nucleus (TRN) subregion consists of GABAergic neurons and controls the activity projecting back into the motor cortex. There are 220 neurons of 8 different cell types with 21 GPCR targets implemented with their own particular coupling to voltage-gated ion channels. As shown experimentally [[33](#_ENREF_33)] in Parkinson’s patients scheduled for deep brain stimulation, the ratio of beta over gamma power of the local field potentials in the STN strongly correlates with hypokinetic symptoms of bradykinesia and rigidity. The calculated ratio of beta/gamma power in our computer model correlates strongly with both the EPS liability in schizophrenia patients with group average data on single antipsychotics for 34 drug-dose combinations and in Parkinson’s patients treated with 22 different therapeutics [[34](#_ENREF_34)].

5. The Cognitive Cortical Network

5.1 Description of the Model

We extended a biophysically realistic model of a network comprised of 20 four-compartment pyramidal cells and 10 two-compartment GABA interneurons [[20](#_ENREF_20), [35](#_ENREF_35)] with the receptor physiology of 18 different dopaminergic, serotonergic, noradrenergic, and cholinergic receptors (Fig 3). Basically this network is a smaller version of the network used for simulating cognitive decline in Alzheimer’s disease [[4](#_ENREF_4)], but with implementation of a different schizophrenia-type pathology.

An mGluR_5_-dependent delayed afterdepolarization current that can increase the spiking rate of pyramidal cells for several seconds was implemented as an alpha function in the model with a time constant similar to the observation in [[24](#_ENREF_24)]. Based on insights from the relative number of pyramidal cells and interneurons [[35](#_ENREF_35), [36](#_ENREF_36)], 40% of the interneurons synapsed with other GABA interneurons, but not with pyramidal cells.

A stimulus is initiated by injecting a current at t=2000 msec which starts the firing of the target pyramidal cells. Without further stimuli, this synchronized firing pattern goes on for a certain amount of time before it gets degraded by the background noise and the interference of the distractor neurons. This time span, called the working memory span, is usually in the range of 4-10 sec and corresponds to the time a certain pattern is held in working memory [[37](#_ENREF_37)].

To calculate this time span, we first divide the time axis in bins of 200 msec and count the number of neurons firing in that time window and determine the time points where this number exceeds M/2, where M is the number of neurons stimulated at t=2000 msec (M=10 for the network). The time difference between these two transition points is the memory span.


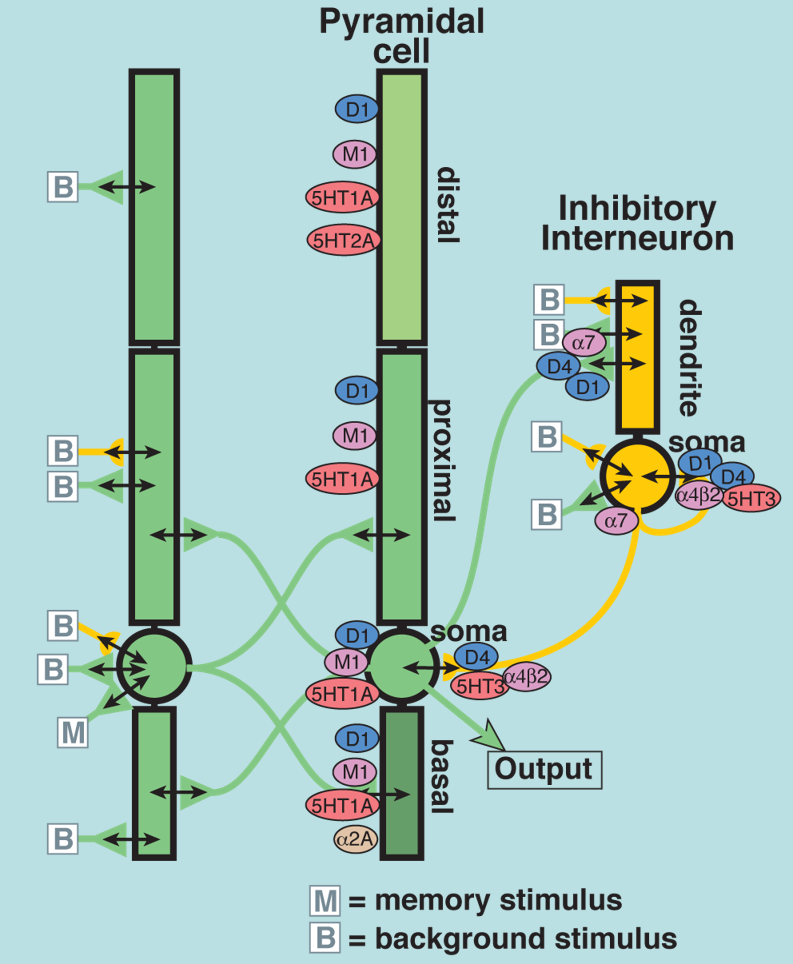


Fig 3. Schematic representation of cortical network model as described in the text

The impact of various GPCR on voltage- or ligand gated ion channels that modulate the firing dynamics of the neuronal network is listed in Table S1

| Receptor | Physiological implementation | Reference |
| --- | --- | --- |
| Dopamine D1R | Increases NMDA, decreases AMPA and increases GABA conductance | [[38](#_ENREF_38)] |
| Dopamine D2R | Presynaptic autoreceptor; modulates AMPAR on pyramidal cells | [[39](#_ENREF_39)] |
| Dopamine D4R | Affects AMPA-R functions | [[40](#_ENREF_40)] |
| Serotonin 5-HT1A | Affects Na-currents | [[41](#_ENREF_41)], [[42](#_ENREF_42), [43](#_ENREF_43)] |
| Serotonin 5-HT1B | Presynaptic autoreceptor in 5-HT synapse | [[43](#_ENREF_43)] |
| Serotonin 5-HT2A | Affects Na and High Threshold Ca-currents | [[44](#_ENREF_44)] |
| Serotonin 5-HT3 | Modulates GABA conductance | [[45](#_ENREF_45)] |
| Serotonin 5-HT4 | Affects serotonergic firing rates; affects K_dr_ and K_s_ and GABA in pyramidal cells | [[46](#_ENREF_46), [47](#_ENREF_47)], |
| Serotonin 5-HT6 | Modulates levels of ACh, NE and DA | [[48](#_ENREF_48)] |
| Adrenergic Alpha2A | Modulates K^+^ conductance on inhibitory interneurons | [[49](#_ENREF_49)] |
| Muscarinic M1R | Cl leak in MSN & K_dr_ in pyramidal cells | [[50](#_ENREF_50)] [[51](#_ENREF_51)] |
| Muscarinic M2R | Presynaptic autoreceptor for cholinergic synapses; | [[52](#_ENREF_52)] [[53](#_ENREF_53)] |
| Nicotinic α7 AChR | Increases presynaptic Glu release, increases interneuron excitability | [[54](#_ENREF_54), [55](#_ENREF_55)] |
| Nicotinic α4β2 AChR | Increases presynaptic GABA release | [[56](#_ENREF_56)] |

Table S2. List of receptors effects implemented in the model, their localization and possible physiological effect. In addition, the model takes into account the physiology of NMDA, AMPA and GABA-A receptors and of mGluR2 although none of the antipsychotics directly interact with these receptors [[54](#_ENREF_54)].

5.2. Implementation of receptor pharmacology

This section deals specifically with the implementation of dopaminergic neurotransmission physiology. Other neuromodulatory processes (serotonergic. cholinergic, noradrenergic) are implemented using similar approaches. In general, we assume a linear normalized relationship between receptor activation and biological effect on physiological responses such as$X_{Y}^{eff}=\frac{X_{Y}^{A}-X_{Y}^{C}}{X_{Y}^{C}}$; where X_Y_^A^ and X_Y_^C^ are the actual activation levels of receptor X subtype Y (for instance D_1_) after treatment (A) and the untreated (placebo) control levels ( C ).

$g_{NMDA}^{*}=g_{NMDA}\times(1+Param_{NMDA}^{D_{1}}\times\frac{D_{1}^{A}-D_{1}^{C}}{D_{1}^{C}})$ Eq S11

$g_{AMPA}^{*}=g_{AMPA}\times(1+Param_{AMPA}^{D_{1}}\times\frac{D_{1}^{A}-D_{1}^{C}}{D_{1}^{C}})$ Eq S12

$g_{GABA}^{*}=g_{GABA}\times(1+Param_{GABA}^{D_{1}}\times\frac{D_{1}^{A}-D_{1}^{C}}{D_{1}^{C}})$ Eq S13

The D_2_S receptor is a presynaptic autoreceptor in the cortex and regulates the activity level of the cortical D_1_ and D_4_ receptors [[57](#_ENREF_57)]. This is taken into account by the cortical dopamine receptor competition model. Furthermore, D_2_R is located postsynaptically on pyramidal cells and modulates EPSP [[39](#_ENREF_39), [58](#_ENREF_58)] through an early AMPA mediated process and a late GABA mediated process. D2R activation modulates AMPA conductance according to

$g_{AMPA}^{*}=g_{AMPA}\times(1+Param_{AMPA}^{D_{2}}\times\frac{D_{2}^{A}-D_{2}^{C}}{D_{2}^{C}})$ Eq S14

where D_2_^A^ and D_2_^C^ are actual D2 activation levels for treated subjects and healthy control levels, respectively and Param_AMPA_^D2^ is an adjustable parameter.

Dopamine D_4_R activation affects AMPA-R similarly but only on interneurons [[40](#_ENREF_40)]. D4R activation increases the AMPA channel conductances such that

$g_{AMPA}^{*}=g_{AMPA}\times(1+Param_{AMPA}^{D_{4}}\times\frac{D_{4}^{A}-D_{4}^{C}}{D_{4}^{C}})$ Eq S15

where D_4_^A^ and D_4_^C^ are actual D2 activation levels for treated subjects and healthy control levels, respectively and Param_AMPA_^D4^ is an adjustable parameter.

5.3. Calibrating the model

First the pathology of schizophrenia is implemented based on the observed changes in patients, a hypodopaminergic tone on the cortical D_1_R [[20](#_ENREF_20)], an NMDA hypofunction [[59](#_ENREF_59), [60](#_ENREF_60)] that is documented by a hypocortical-hyperstriatal imbalance in metabolic imaging [[61](#_ENREF_61)], a GABA deficit more specifically in the chandelier interneurons [[62](#_ENREF_62)] but applied here to the interneurons in the network, and a more noisy background signal [[63](#_ENREF_63)]. This leads to a cognitive deficit in schizophrenia patients which is dependent upon the clinical readout, but on average is 1.5 standard deviations lower than healthy controls [[64-66](#_ENREF_64)].

We first determine the numerical changes in these four parameters that will define the “schizophrenia” state of the network. For this, we simulate the effect of 100 different virtual ‘normal subjects’ that have variable values around the optimal values. Based on the observation that schizophrenia patients perform at a level that is about 1.5 standard deviations lower than healthy controls on a number of cognitive tests, we can then determine the minimal changes in the four parameters that corresponds to a network cognitive outcome as determined by this difference.

In a second step, the remaining free biological coupling factors such as Param_AMPA_^D4^ are further calibrated using clinical data on the N-back working memory test as reported in schizophrenia patients, healthy controls with anticholinergics or COMT inhibitors and stratified according to the COMT genotype. Table 1 lists the clinical observations on diverse interventions used for calibrating the cortical cognitive network.

| Subjects | Treatment | COMT genotype | 2-Back WM performance (% correct) |
| --- | --- | --- | --- |
| Schizophrenia | Placebo | COMT Met/Met | 49.8 |
| Schizophrenia | Placebo | COMT Val/Met | 42 |
| Schizophrenia | Placebo | COMT Val/Val | 43.4 |
| Schizophrenia | Perphenazine 8, Ziprasidone 80  Quetiapine 600 | COMT Met/Met | 61.6 |
| Schizophrenia | Clozapine 250, Haldol 10, Quetiapine 700, Risperidone 6 | COMT Val/Met | 40.1 |
| Schizophrenia | Haldol 10, Risperidone 6 | COMT Val/Val | 43.6 |
| Schizophrenia | Olanzapine 20 | COMT Met/Met | 73 |
| Schizophrenia | Olanzapine 20 | COMT Val/Met | 56.6 |
| Schizophrenia | Olanzapine 20 | COMT Val/Val | 47.6 |
| Healthy volunteers | Placebo | N/A, assumed to be heterozygous | 81 |
| Healthy volunteers | Mecamylamine | N/A assumed to be heterozygous | 79 |
| Healthy volunteers | Scopolamine | N/A assumed to be heterozygous | 69 |
| Healthy volunteers | Mecamylamine & scopolamine | N/A assumed to be heterozygous | 61 |
| Healthy volunteers | Placebo | COMT Met/Met | 83 |
| Healthy volunteers | Tolcapone | COMT Met/Met | 82 |
| Healthy volunteers | Placebo | COMT Val/Val | 76 |
| Healthy volunteers | Placebo | COMT Val/Val | 81 |

Table S2. Clinical changes in 2-back working memory performances for a number of therapeutic interventions. The data are taken from studies in schizophrenia patients [[67](#_ENREF_67), [68](#_ENREF_68)] and healthy controls [[69](#_ENREF_69)] with cholinergic changes and with COMT modulation [[70](#_ENREF_70)]. For experiments where no COMT genotype data are available, we assume a COMT MV genotype.

The calibration of the network is performed using ‘Design of Experiment’ (DOE) statistical techniques, rather than OFAT (one Factor At a Time). OFAT techniques, besides being computationally intensive also are unable to detect interaction between parameters to be calibrated [[71](#_ENREF_71)]. DOE techniques are computationally effective and provide a sound statistical approach to identify the driving parameters.

A good robust approach uses 2n simulations, where n is the number of free parameters, compared to 2^n^ for a full OFAT design [[72](#_ENREF_72)] .

A [2n x n] matrix A is then constructed with elements a_ij_, where i is the run number (1..14) and j = the calibration parameter (1..7) and defined by

a_ij_ = Max(j) if e_ij_ = ’P’ and a_ij_ = Min(j) if e_ij_ = ’M’ Eq S16

An [n x 1] row Average+ is constructed with elements Average+_j_ =$\sum_{i=1}^{14} Outcome[i]\times Pos_{ij}$

where Pos_ij_ = 1 if e_ij_ = ’P’ and Pos_ij_ = 0 if e_ij_ = ’M’ Eq S17

Similarly, an [n x 1] row Average- is constructed with elements

Average-_j_ =$\sum_{i=1}^{14} Outcome[i]\times Neg_{ij}$ Eq S18

where Neg_ij_ = 0 if e_ij_ = ’P’ and Neg_ij_ = 1 if e_ij_ = ’M’ Eq S19

The Pareto-effect, Par_j_ (j=1..n), is simply the difference Average+_j_ - Average-_j_ and indicates both the strength and the sign of the gradient towards the optimum.

The next iteration will use this information to adjust the range of the parameters, until the Pareto effects become so small that a more detailed surface response is initiated.

1. The concept of information bandwidth

Recent studies have indicated that complex encoding in the human brain is not only driven by firing frequency of neurons but by an “information bandwidth” akin to the Shannon entropy measure [[73](#_ENREF_73)]. Similarly we implemented this readout based on the extrapolation first proposed by [[74](#_ENREF_74)] and further described in [[32](#_ENREF_32)].

Basically, the spike train is discretized into time bins with size ΔT. For the analysis of information content, the full spike train is divided in segments with a length T, so that each possible neural response is a word with T/ΔT symbols, where ΔT is 1, 2, 3 or 4 msec (see below). A bin with an action potential is given the value 1, otherwise it is 0. In this way, words can be defined with maximal length T/ΔT. This ratio is 4, 8, 12 and 16, so that maximal word length spans a time segment between 4 and 64 msec.

Using the sliding window approach as shown in Fig 2 we calculate a distribution of words and derive the normalized count p_i_ of i^th^ word [i=1, 2, 3, …, 2^16^-1] so that a naïve estimate of the entropy is given by S _naïve_ (T, ΔT, size) = Σ p_i_  log (p_i_). True entropy is independent of data set size and is reached when the size goes to infinity such that S(T, ΔT)=lim _size →∞_ S _naïve_ (T, ΔT, size). 

We calculate the values for S _naïve_ (T, ΔT, size), where size = total duration of the spike train, and i is restricted by the maximal word length of 16. We get a parabolic estimate of the total entropy S(T, ΔT) by fitting the following equation from our calculated entropy at 3 different action potential train sizes, 8000, 10000 and 12000 msec,

S _naïve_ (T, ΔT, size) = S(T, ΔT) + S_1_(T, ΔT)/size + S_2_(T, ΔT)/size^2^ (Eq S20)

This results in a set of 3 equations with x_i_=1/size_i_ and with y_i_ the calculated naive entropy values such that y_i_ (j) =A+Bx_i_(j) + Cx_i_(j)^2^ with j=1,2,3, giving us a value for the intercept A or S(T, ΔT).

The real entropy is independent of the amount of time; therefore we are interested in the entropy change over time in bits/sec that can be calculated as S(Δ T)=lim _T →∞_ S(T,ΔT)/T.

Plotting the total entropy S(T,Δ T) as a function of 1/T results in the intercept at the x-axis representing the real entropy change. Studies of the entropy estimation in the undersampled limit (i.e. total finite experiment duration, in this case 2500 msec) suggest that for T values less than 100 msec, the lower bound of the entropy and the naive estimate are only 10-15% apart [[74](#_ENREF_74)].


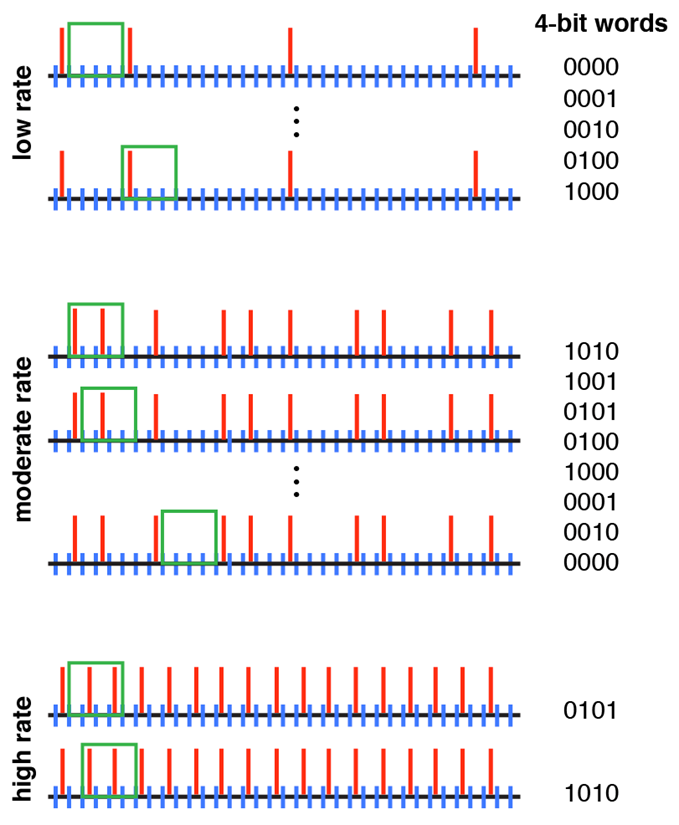


Figure S4 Schematic representation of the concept of information content. First the activity of all neurons in a specific region is projected on the time axis; for each time bin, an action potential generates a value of 1, otherwise zero. Bin time windows are defined such that at most one action potential is present. At low firing rates, information content is limited (in this example only five different 4-bit words can be formed). When the firing increases to a moderate rate, the information content increases as there are many more possible combinations of sequences present (in this example 8 different 4-bit words), because of a large variability of interspike interval. When the firing increases almost to saturation (in this case every other bin), the information content decreases to two 4-bit words, because there are not as many possible combinations (interspike interval driven by refractory period of neurons). The Shannon entropy principle is based on the product of p times ln(p), where p is the probability of any given 4-bit word.

References

1. Spiros, A., R. Carr, and H. Geerts, *Not all partial dopamine D(2) receptor agonists are the same in treating schizophrenia. Exploring the effects of bifeprunox and aripiprazole using a computer model of a primate striatal dopaminergic synapse.* Neuropsychiatr Dis Treat, 2010. **6**: p. 589-603.

2. Montague, P.R., et al., *Dynamic gain control of dopamine delivery in freely moving animals.* J Neurosci, 2004. **24**(7): p. 1754-9.

3. Geerts, H., *alpha7 Nicotinic receptor modulators for cognitive deficits in schizophrenia and Alzheimer's disease.* Expert Opin Investig Drugs, 2012. **21**(1): p. 59-65.

4. Roberts, P.D., A. Spiros, and H. Geerts, *Simulations of symptomatic treatments for Alzheimer's disease: computational analysis of pathology and mechanisms of drug action.* Alzheimers Res Ther, 2012. **4**(6): p. 50.

5. Spiros, A. and L. Edelstein-Keshet, *Testing a model for the dynamics of actin structures with biological parameter values.* Bull Math Biol, 1998. **60**(2): p. 275-305.

6. Xiberas, X., et al., *Extrastriatal and striatal D(2) dopamine receptor blockade with haloperidol or new antipsychotic drugs in patients with schizophrenia.* Br J Psychiatry, 2001. **179**: p. 503-8.

7. Slifstein, M., et al., *COMT genotype predicts cortical-limbic D1 receptor availability measured with [11C]NNC112 and PET.* Mol Psychiatry, 2008. **13**(8): p. 821-7.

8. Pirini, M., et al., *A computational modelling approach to investigate different targets in deep brain stimulation for Parkinson's disease.* J Comput Neurosci, 2009. **26**(1): p. 91-107.

9. Bazhenov, M., et al., *Computational models of thalamocortical augmenting responses.* J Neurosci, 1998. **18**(16): p. 6444-65.

10. Rubin, J.E. and D. Terman, *High frequency stimulation of the subthalamic nucleus eliminates pathological thalamic rhythmicity in a computational model.* J Comput Neurosci, 2004. **16**(3): p. 211-35.

11. Hodgkin, A.L. and A.F. Huxley, *A quantitative description of membrane current and its application to conduction and excitation in nerve.* J Physiol, 1952. **117**(4): p. 500-44.

12. Spiros, A., P. Roberts, and H. Geerts, *A Quantitative Systems Pharmacology Computer Model for Schizophrenia Efficacy and Extrapyramidal Side Effects.* Drug Development Research, 2012. **73:** (4): p. 196-213.

13. Falk, T., et al., *Over-expression of the potassium channel Kir2.3 using the dopamine-1 receptor promoter selectively inhibits striatal neurons.* Neuroscience, 2008. **155**(1): p. 114-27.

14. Gruber, A.J., et al., *Modulation of striatal single units by expected reward: a spiny neuron model displaying dopamine-induced bistability.* J Neurophysiol, 2003. **90**(2): p. 1095-114.

15. Kuzhikandathil, E.V. and G.S. Oxford, *Classic D1 dopamine receptor antagonist R-(+)-7-chloro-8-hydroxy-3-methyl-1-phenyl-2,3,4,5-tetrahydro-1H-3-benzaze pine hydrochloride (SCH23390) directly inhibits G protein-coupled inwardly rectifying potassium channels.* Mol Pharmacol, 2002. **62**(1): p. 119-26.

16. Mermelstein, P.G., et al., *Inwardly rectifying potassium (IRK) currents are correlated with IRK subunit expression in rat nucleus accumbens medium spiny neurons.* J Neurosci, 1998. **18**(17): p. 6650-61.

17. Hernandez-Lopez, S., et al., *D2 dopamine receptors in striatal medium spiny neurons reduce L-type Ca2+ currents and excitability via a novel PLC[beta]1-IP3-calcineurin-signaling cascade.* J Neurosci, 2000. **20**(24): p. 8987-95.

18. Bargas, J., et al., *Cellular and molecular characterization of Ca2+ currents in acutely isolated, adult rat neostriatal neurons.* J Neurosci, 1994. **14**(11 Pt 1): p. 6667-86.

19. Geerts, H., P. Roberts, and A. Spiros, *A quantitative system pharmacology computer model for cognitive deficits in schizophrenia.* CPT Pharmacometrics Syst Pharmacol, 2013. **2**: p. e36.

20. Durstewitz, D., J.K. Seamans, and T.J. Sejnowski, *Dopamine-mediated stabilization of delay-period activity in a network model of prefrontal cortex.* J Neurophysiol, 2000. **83**(3): p. 1733-50.

21. Destexhe, A. and D. Pare, *Impact of network activity on the integrative properties of neocortical pyramidal neurons in vivo.* J Neurophysiol, 1999. **81**(4): p. 1531-47.

22. Spruston, N., D.B. Jaffe, and D. Johnston, *Dendritic attenuation of synaptic potentials and currents: the role of passive membrane properties.* Trends Neurosci, 1994. **17**(4): p. 161-6.

23. Larkman, A.U., *Dendritic morphology of pyramidal neurones of the visual cortex of the rat: III. Spine distributions.* J Comp Neurol, 1991. **306**(2): p. 332-43.

24. Sidiropoulou, K., et al., *Dopamine modulates an mGluR5-mediated depolarization underlying prefrontal persistent activity.* Nat Neurosci, 2009. **12**(2): p. 190-9.

25. Williams, G.V. and P.S. Goldman-Rakic, *Modulation of memory fields by dopamine D1 receptors in prefrontal cortex.* Nature, 1995. **376**(6541): p. 572-5.

26. Nicholas, T., , Sridhar Duvvuri, Claire Leurent, David Raunig, Tracey Rapp, Phil Iredale , Carolyn Rowinski, Robert Carr, Patrick Roberts, Athan Spiros, Hugo Geerts, *Systems Pharmacology Modeling in Neuroscience: Prediction and Outcome of PF-04995274, a 5HT4 Partial Agonist, in a Clinical Scopolamine Impairment Trial* Advances in Alzheimer's Disease, 2013. **2**(3): p. 83-98.

27. Geerts, H., Spiros A, Roberts P, *Assessing the synergy between cholinomimetics and memantine as augmentation therapy in Cognitive Impairment in Schizophrenia. A virtual human patient trial using Quantitative Systems Pharmacology.* Frontiers in Pharmacology, 2015. **6**: p. 198.

28. Geerts, H., et al., *Understanding responder neurobiology in schizophrenia using a quantitative systems pharmacology model: Application to iloperidone.* J Psychopharmacol, 2015. **29**(4): p. 372-82.

29. Traub, R.D., et al., *Cellular mechanisms of neuronal population oscillations in the hippocampus in vitro.* Annu Rev Neurosci, 2004. **27**: p. 247-78.

30. Destexhe, A., et al., *Fluctuating synaptic conductances recreate in vivo-like activity in neocortical neurons.* Neuroscience, 2001. **107**(1): p. 13-24.

31. Kita, H., *Balance of Monosynaptic Excitatory and Disynaptic Inhibitory Responses of the Globus Pallidus Induced after Stimulation of the Subthalamic Nucleus in the Monkey.* Journal of Neuroscience, 2005. **25**(38): p. 8611-8619.

32. Spiros, A., P. Roberts, and H. Geerts, *Semi-mechanistic computer simulation of psychotic symptoms in schizophrenia with a model of a humanized cortico-striatal-thalamocortical loop.* European neuropsychopharmacology : the journal of the European College of Neuropsychopharmacology, 2017. **27**(2): p. 107-119.

33. Little, S., et al., *Adaptive deep brain stimulation in advanced Parkinson disease.* Ann Neurol, 2013. **74**(3): p. 449-57.

34. Roberts, P., A. Spiros, and H. Geerts, *A Humanized clinically calibrated quantitative systems pharmacology model for hypokinetic motor symptoms in Parkinson's Disease.* Frontiers in Pharmacology, 2016. **7**( ): p. 6.

35. DeFelipe, J., *Cortical interneurons: from Cajal to 2001.* Prog Brain Res, 2002. **136**: p. 215-38.

36. Isaacson, J.S. and M. Scanziani, *How inhibition shapes cortical activity.* Neuron, 2011. **72**(2): p. 231-43.

37. Levy, R. and P.S. Goldman-Rakic, *Segregation of working memory functions within the dorsolateral prefrontal cortex.* Exp Brain Res, 2000. **133**(1): p. 23-32.

38. Law-Tho, D., J.C. Hirsch, and F. Crepel, *Dopamine modulation of synaptic transmission in rat prefrontal cortex: an in vitro electrophysiological study.* Neurosci Res, 1994. **21**(2): p. 151-60.

39. Tseng, K.Y. and P. O'Donnell, *D2 dopamine receptors recruit a GABA component for their attenuation of excitatory synaptic transmission in the adult rat prefrontal cortex.* Synapse, 2007. **61**(10): p. 843-50.

40. Yuen, E.Y. and Z. Yan, *Dopamine D4 receptors regulate AMPA receptor trafficking and glutamatergic transmission in GABAergic interneurons of prefrontal cortex.* J Neurosci, 2009. **29**(2): p. 550-62.

41. Foehring, R.C., *Serotonin modulates N- and P-type calcium currents in neocortical pyramidal neurons via a membrane-delimited pathway.* J Neurophysiol, 1996. **75**(2): p. 648-59.

42. Cardenas, C.G., L.P. Del Mar, and R.S. Scroggs, *Two parallel signaling pathways couple 5HT1A receptors to N- and L-type calcium channels in C-like rat dorsal root ganglion cells.* J Neurophysiol, 1997. **77**(6): p. 3284-96.

43. Gerhardt, C.C. and H. van Heerikhuizen, *Functional characteristics of heterologously expressed 5-HT receptors.* Eur J Pharmacol, 1997. **334**(1): p. 1-23.

44. Carr, D.B., et al., *Serotonin receptor activation inhibits sodium current and dendritic excitability in prefrontal cortex via a protein kinase C-dependent mechanism.* J Neurosci, 2002. **22**(16): p. 6846-55.

45. Puig, M.V., et al., *In vivo excitation of GABA interneurons in the medial prefrontal cortex through 5-HT3 receptors.* Cereb Cortex, 2004. **14**(12): p. 1365-75.

46. Ansanay, H., et al., *cAMP-dependent, long-lasting inhibition of a K+ current in mammalian neurons.* Proc Natl Acad Sci U S A, 1995. **92**(14): p. 6635-9.

47. Cai, X., et al., *Activity-dependent bidirectional regulation of GABA(A) receptor channels by the 5-HT(4) receptor-mediated signalling in rat prefrontal cortical pyramidal neurons.* J Physiol, 2002. **540**(Pt 3): p. 743-59.

48. Riemer, C., et al., *Influence of the 5-HT6 receptor on acetylcholine release in the cortex: pharmacological characterization of 4-(2-bromo-6-pyrrolidin-1-ylpyridine-4-sulfonyl)phenylamine, a potent and selective 5-HT6 receptor antagonist.* J Med Chem, 2003. **46**(7): p. 1273-6.

49. Boehm, S., *Presynaptic alpha2-adrenoceptors control excitatory, but not inhibitory, transmission at rat hippocampal synapses.* J Physiol, 1999. **519 Pt 2**: p. 439-49.

50. Perez-Rosello, T., et al., *Cholinergic control of firing pattern and neurotransmission in rat neostriatal projection neurons: role of CaV2.1 and CaV2.2 Ca2+ channels.* J Neurophysiol, 2005. **93**(5): p. 2507-19.

51. Shen, W., et al., *Cholinergic suppression of KCNQ channel currents enhances excitability of striatal medium spiny neurons.* J Neurosci, 2005. **25**(32): p. 7449-58.

52. Parnas, H., et al., *Depolarization initiates phasic acetylcholine release by relief of a tonic block imposed by presynaptic M2 muscarinic receptors.* J Neurophysiol, 2005. **93**(6): p. 3257-69.

53. Zhang, W., et al., *Multiple muscarinic acetylcholine receptor subtypes modulate striatal dopamine release, as studied with M1-M5 muscarinic receptor knock-out mice.* J Neurosci, 2002. **22**(15): p. 6347-52.

54. Parikh, V., et al., *Prefrontal beta2 subunit-containing and alpha7 nicotinic acetylcholine receptors differentially control glutamatergic and cholinergic signaling.* J Neurosci, 2010. **30**(9): p. 3518-30.

55. Alkondon, M. and E.X. Albuquerque, *Nicotinic acetylcholine receptor alpha7 and alpha4beta2 subtypes differentially control GABAergic input to CA1 neurons in rat hippocampus.* J Neurophysiol, 2001. **86**(6): p. 3043-55.

56. Aracri, P., et al., *Tonic modulation of GABA release by nicotinic acetylcholine receptors in layer V of the murine prefrontal cortex.* Cereb Cortex, 2010. **20**(7): p. 1539-55.

57. Tamminga, C.A. and A. Carlsson, *Partial dopamine agonists and dopaminergic stabilizers, in the treatment of psychosis.* Curr Drug Targets CNS Neurol Disord, 2002. **1**(2): p. 141-7.

58. Sun, X., Y. Zhao, and M.E. Wolf, *Dopamine receptor stimulation modulates AMPA receptor synaptic insertion in prefrontal cortex neurons.* J Neurosci, 2005. **25**(32): p. 7342-51.

59. Moghaddam, B., *Recent basic findings in support of excitatory amino acid hypotheses of schizophrenia.* Prog Neuropsychopharmacol Biol Psychiatry, 1994. **18**(5): p. 859-70.

60. Coyle, J. and G. Tsai, *The NMDA receptor glycine modulatory site: a therapeutic target for improving cognition and reducing negative symptoms in schizophrenia.* Psychopharmacology, 2003. **174**(1).

61. Meyer-Lindenberg, A., et al., *Reduced prefrontal activity predicts exaggerated striatal dopaminergic function in schizophrenia.* Nat Neurosci, 2002. **5**(3): p. 267-71.

62. Volk, D.W. and D.A. Lewis, *Impaired prefrontal inhibition in schizophrenia: relevance for cognitive dysfunction.* Physiol Behav, 2002. **77**(4-5): p. 501-5.

63. Winterer, G., et al., *Schizophrenia: reduced signal-to-noise ratio and impaired phase-locking during information processing.* Clin Neurophysiol, 2000. **111**(5): p. 837-49.

64. Saykin, A.J., et al., *Neuropsychological deficits in neuroleptic naive patients with first-episode schizophrenia.* Arch Gen Psychiatry, 1994. **51**(2): p. 124-31.

65. Kurtz, M.M., et al., *Approaches to cognitive remediation of neuropsychological deficits in schizophrenia: a review and meta-analysis.* Neuropsychol Rev, 2001. **11**(4): p. 197-210.

66. Elvevag, B. and T.E. Goldberg, *Cognitive impairment in schizophrenia is the core of the disorder.* Crit Rev Neurobiol, 2000. **14**(1): p. 1-21.

67. Bertolino, A., et al., *Interaction of COMT (Val(108/158)Met) genotype and olanzapine treatment on prefrontal cortical function in patients with schizophrenia.* Am J Psychiatry, 2004. **161**(10): p. 1798-805.

68. Weickert, T.W., et al., *Catechol-O-methyltransferase val108/158met genotype predicts working memory response to antipsychotic medications.* Biol Psychiatry, 2004. **56**(9): p. 677-82.

69. Green, A., et al., *Muscarinic and nicotinic receptor modulation of object and spatial n-back working memory in humans.* Pharmacol Biochem Behav, 2005. **81**(3): p. 575-84.

70. Roussos, P., S.G. Giakoumaki, and P. Bitsios, *Tolcapone effects on gating, working memory, and mood interact with the synonymous catechol-O-methyltransferase rs4818c/g polymorphism.* Biol Psychiatry, 2009. **66**(11): p. 997-1004.

71. Box, H.C., E.E. Budzinski, and H.G. Freund, *Studies of electrons trapped in X-irradiated rhamnose crystals.* Radiat Res, 1990. **121**(3): p. 262-6.

72. Watts, A., M. Watts, and J. Snelling, *Seeds of genius : the early writings of Alan Watts*1997, Shaftesbury, Dorset ; Rockport, Mass: Element Books. viii, 312 p.

73. Pryluk, R., et al., *A Tradeoff in the Neural Code across Regions and Species.* Cell, 2019. **176**(3): p. 597-609 e18.

74. Strong, S.P., et al., *On the application of information theory to neural spike trains.* Pac Symp Biocomput, 1998: p. 621-32.
